# Dual enzyme-driven redox homeostasis disruption with ultrasmall Pt-decorated MoS_2_ for combined ferroptosis therapy of hepatocellular carcinoma

**DOI:** 10.1016/j.mtbio.2025.102260

**Published:** 2025-08-30

**Authors:** Mengmeng Dong, Yimo Wang, Lidong Cao, Xiaobin Fei, Junjie Qian, Qingqing Wu, Lu Zhou, Yueqin Zhang, Wei Duan, Chengwu Zhang, Changwei Dou

**Affiliations:** a General Surgery, Cancer Center, Department of Hepatobiliary & Pancreatic Surgery and Minimally Invasive Surgery, Zhejiang Provincial People's Hospital, Affiliated People's Hospital, Hangzhou Medical College, Hangzhou, Zhejiang, China; b Clinical Research Institute, Zhejiang Provincial People's Hospital, Affiliated People's Hospital, Hangzhou Medical College, Hangzhou, Zhejiang, China; c Department of Surgery, Hangzhou Normal University Second Clinical Medical College, Zhejiang Provincial People's Hospital, Hangzhou, Zhejiang, China; d Department of Radiation Oncology, Tohoky University, Sendai, Japan; e School of Pharmacy, Hangzhou Normal University, Hangzhou, Zhejiang, China

**Keywords:** Ferroptosis therapy, Nanozyme, Hepatocellular carcinoma, Tumor microenvironment, Photothermal therapy

## Abstract

There is an urgent clinical task for developing novel and precise therapies to enhance the treatment efficiency of hepatocellular carcinoma (HCC). Ferroptosis, a form of non-apoptotic regulated cell death, plays a significant role in improving cancer treatment outcomes. However, the robust antioxidant defense system within the tumor microenvironment (TME) severely impacts the ferroptosis efficacy. Nanozymes are emerging as promising candidates for inducing ferroptosis, but constructing novel nanozymes with high catalytic efficiency and multiple activities to amplify ferroptosis's therapeutic effects remains challenging. Here, we have judiciously fabricated a bimetallic nanozyme of ultrasmall Pt-decorated MoS_2_ (Pt@MoS_2_) through a simple and efficient aqueous synthesis strategy. The doping of ultrasmall Pt nanoparticles significantly enhances the photothermal activity, peroxidase-like activity, and glutathione oxidase-like activity of MoS_2_. Under the weak acidic conditions, over-expressed hydrogen peroxide (H_2_O_2_), and high glutathione (GSH) levels, Pt@MoS_2_ nanozyme with dual enzyme-driven redox homeostasis disruption can be activated to break down the antioxidant defense system and reshape the TME, effectively causing ·OH production from H_2_O_2_ and GSH consumption. The dual nanozyme-remodulated TME suffers from oxidative cellular damage and significantly accelerates HCC apoptosis and ferroptosis. Both *in vitro* and *in vivo* experimental results demonstrate that Pt@MoS_2_ has successfully induced cancer cell oxidative damage, lipid peroxidation, and downregulation of glutathione peroxidase 4 expression under near-infrared (NIR) laser-assisted photothermal therapy, exhibiting remarkable antitumor efficacy and good biosafety. This study offers insights into designing efficient bimetallic nanozymes for enhanced TME reshaping and ferroptosis therapy and expanding nanozymes' application in cancer treatment.

## Highlights

• Ultrasmall Pt-decorated MoS_2_ (Pt@MoS_2_) was created through a simple and efficient aqueous synthesis strategy.
• Pt@MoS_2_ shows significantly enhanced photothermal activity, POD-like activity, and GSHOx-like activity.
• Pt@MoS_2_ induced redox homeostasis disruption under NIR laser for ferroptosis amplification of HCC.
• This work provides valuable insights into the structure-activity relationship of nanozymes for tumor combined therapy.

## Graphical abstract

**Figure:**
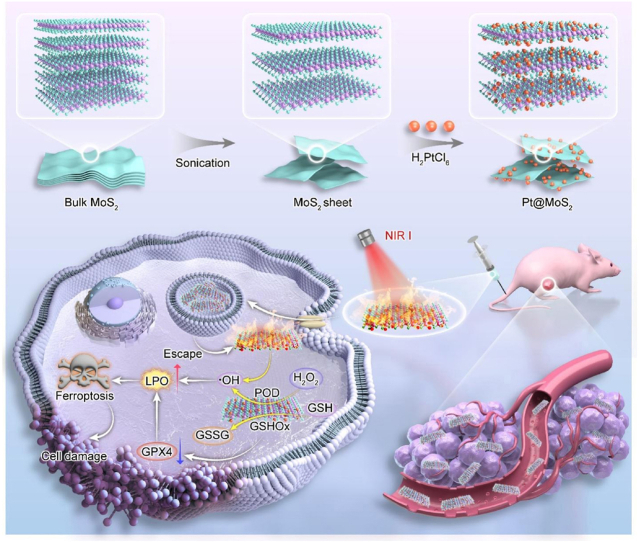

## 1 Introduction

Hepatocellular carcinoma (HCC) is one of the most common malignant tumors globally and has become the third leading cause of cancer-related deaths [1,2]. Notably, HCC is characterized by strong heterogeneity and insidious onset, resulting in most HCC patients being diagnosed at an advanced stage [3]. These patients thus miss the opportunity for surgical resection, leading to an increasing number of annual deaths and new cases [4]. Traditional HCC treatment methods such as surgery, radiotherapy, and chemotherapy face issues of high metastasis and recurrence rates in clinical treatment [[5], [6], [7]]. Moreover, the effectiveness of tumor treatment largely depends on the tumor microenvironment (TME) [7,8]. The TME is known for various characteristics, such as weak acidity, hypoxia, high levels of reactive oxygen species (ROS), and high concentrations of glutathione (GSH) [[9], [10], [11]]. It has been reported that the TME can not only protect cancer cells from drug attacks and suppress antitumor effects, but also induce tumor proliferation and metastasis, directly relating to the effectiveness of treatment and the formation of resistance [12,13].

Excitingly, ferroptosis, as a form of non-apoptotic regulated cell death pathway closely related to the TME, has been proven to play a significant role in enhancing cancer treatment outcomes [14,15]. Recent studies have indicated that cancer cells resistant to traditional therapies or with high metastatic tendencies are particularly susceptible to ferroptosis [[15], [16], [17]]. Ferroptosis is characterized by the lethal accumulation of iron-dependent membrane-localized lipid peroxides, accompanied by the dysregulation of GSH metabolism and the production of excessive intracellular ROS, as well as the inactivation of glutathione peroxidase 4 (GPX4) [18]. However, the robust antioxidant defense system within the TME significantly impacts the therapeutic efficacy of ferroptosis. It has been reported that increasing the intracellular ROS levels in tumor cells and depleting GSH within tumor tissues can effectively enhance the efficacy of ferroptosis [19,20]. Therefore, the pursuit of precise therapeutic interventions that modulate the TME and target ferroptosis is crucial for improving the clinical management of HCC. These interventions are intended to enhance treatment efficacy, mitigate side effects, and avert the onset of multidrug resistance.

In recent years, the emergence of nanozymes has provided an ideal choice for constructing novel ferroptosis inducers that regulate the TME [21,22]. Nanozymes are a class of biomimetic nanocatalysts with high catalytic efficiency and inherent enzyme-like characteristics [23,24]. Due to their adjustable catalytic activity, high stability, ease of preparation, and low cost, nanozymes have been widely applied in various biomedical fields, such as molecular diagnostics, targeted delivery, disease treatment, and bioimaging [[25], [26], [27]]. These advanced nanozymes possess the capacity to manifest a range of activities, including peroxidase (POD), catalase (CAT), superoxide dismutase (SOD), and glutathione oxidase (GSHOx), thereby facilitating effective cancer treatment through direct modulation of the TME [[28], [29], [30]]. In particular, nanozymes endowed with the capacity to regulate ROS and GSH have garnered considerable attention in ferroptosis-based therapeutic strategies. Specifically, the POD-like nanozymes can catalyze the decomposition of H_2_O_2_ to produce hydroxyl radicals (·OH), thereby increasing the intracellular ROS levels and promoting the accumulation of lipid peroxidation (LPO) to induce ferroptosis [31]. On the other hand, nanozymes with GSHOx mimetic activity can convert overexpressed GSH within TME into its oxidized form, glutathione disulfide (GSSG) [32]. This GSH depletion further leads to downregulation of GPX4 expression and enhanced accumulation of LPO, thereby effectively inducing ferroptosis [33]. Currently, numerous TME-responsive nanozymes with redox homeostasis disruption have been developed, including noble metal nanomaterials, metal oxides/sulfides, and metal-organic framework materials [[33], [34], [35], [36]]. However, the majority of nanozymes exhibit limited catalytic activity and specificity within the highly complex TME. The development of multifunctional nanozyme with superior catalytic performance and redox homeostasis disruption for enhancing TME-responsive ferroptosis therapeutic effects is highly needed but challenging.

Inspired by the natural enzyme cofactor stimulation mechanism, enhancing the catalytic performance of nanozymes through heteroatom doping has attracted increasing interest from researchers [[37], [38], [39]]. Metallic doping can endow the original nanocatalyst with some special properties, such as changes in size and structure, increase in dispersibility, and the introduction of novel catalytic sites [40,41]. Among numerous metals, Pt nanoparticles (NPs) have received widespread attention due to their excellent catalytic performance and unique optical properties [[42], [43], [44]].Constructing Pt-based hybrid nanozymes can effectively avoid the aggregation of high-surface-energy Pt NPs, reduce the amount of Pt used, and improve the benefit-to-risk ratio [45,46]. In the past few years, Pt-based hybrid nanozymes have flourished and achieved significant progress in tumor combination therapy. For example, Yang et al. prepared a PtSn bimetallic nanocluster (Pt size: ∼2 nm) with enhanced POD-like and CAT-like activity using a high-temperature solvothermal method [47]. Benefiting from the active center doping of ultrasmall-sized Pt and oxygen vacancies of Sn, the PtSn bimetallic nanozyme possesses high catalytic activities, which can significantly enhance dual-enzyme activity and photothermal characteristics for enhanced tumor photothermal/catalytic combination therapy. Therefore, due to the synergistic action of metal sites, the rational design of Pt-based bimetallic nanozymes is expected to enhance the treatment effects on tumors [48]. However, to our knowledge, there have been few studies on Pt-based bimetallic nanozymes with dual ROS and GSH regulatory functions for tumor ferroptosis amplification. Moreover, the preparation processes of most Pt-based nanozymes require high temperature, high pressure, and organic solvent reaction conditions [45,49]. Therefore, the development of novel Pt-based metallic hybrid nanozymes through a simple and mild synthesis strategy for reshaping the TME steady state and amplifying the ferroptosis-dependent combination therapeutic effects still needs further exploration.

**Scheme 1:**
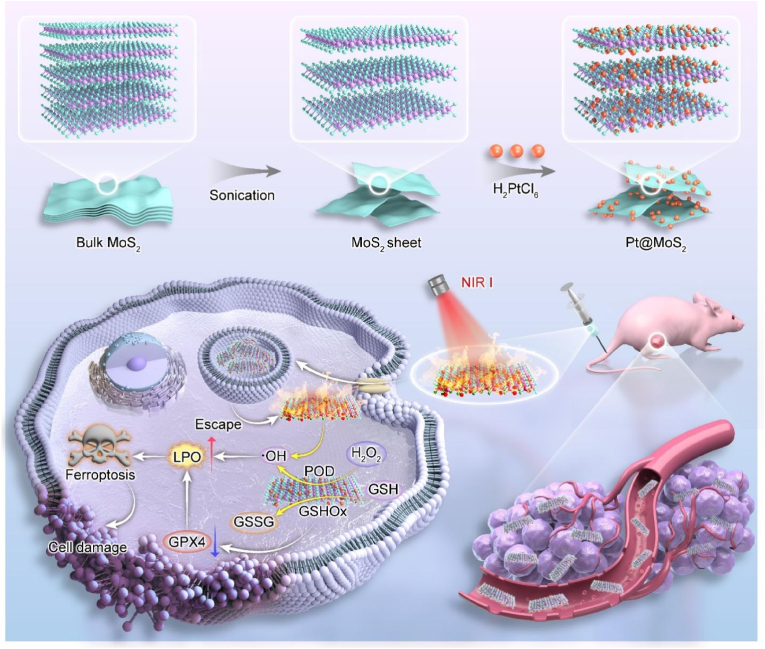
Schematic representation for the preparation process of ultrasmall Pt-decorated MoS_2_ nanocatalyst and their application for NIR photothermal-enhanced ferroptosis therapy of hepatocellular carcinoma.

As a proof of concept, in this study, we constructed an ultrasmall Pt-decorated bimetallic hybrid nanozyme (Pt@MoS_2_) with POD and GSHOx dual-enzyme-like activity, which can effectively regulate the levels of ROS and GSH in the TME for efficient photothermal/catalytic combination therapy of HCC tumors dependent on the ferroptosis pathway (Scheme 1). The Pt@MoS_2_ bimetallic hybrid nanozyme was prepared through a two-step strategy in the aqueous phase. Firstly, thin-layer MoS_2_ nanosheets were obtained from MoS_2_ powder through a classic polyvinyl pyrrolidone (PVP) assisted liquid-phase exfoliation method. Subsequently, by leveraging the self-reduction reaction between MoS_2_ and Pt and the constraining effect of PVP, Pt@MoS_2_ composite NPs were successfully obtained in the aqueous phase. As a TME-responsive nanozyme, Pt@MoS_2_ exhibits significantly increased POD-like and GSHOx-like activities compared to the MoS_2_ precursor under acidic conditions. Moreover, to better understand the catalytic reactions of the bimetallic nanozyme, theoretical calculations were employed to reveal the catalytic process of POD-like catalytic activity, for the first time confirming the potential mechanism of Pt doping to improve the catalytic effect of single MoS_2_. Importantly, Pt@MoS_2_ also demonstrates excellent photothermal performance under near-infrared (NIR) laser irradiation. Based on the outstanding catalytic/photothermal performance of Pt@MoS_2_ nanozyme, the dual enzyme-driven redox homeostasis disruption propels tumor cells into a perpetual cycle of oxidative stress disequilibrium, which further leads to extensive lipid peroxidation and the induction of ferroptosis in tumor cells under NIR laser assistance, exhibiting remarkable antitumor efficacy and good biosafety. Overall, this study not only provides new insights into the rational design of efficient bimetallic nanozymes with TME regulatory effects but also offers a new strategy for clinical HCC tumor treatment based on ferroptosis.

## 2 Results and discussion

### 2.1 Synthesis and characterization

In this study, the synthesis of Pt@MoS_2_ nanozyme involves a two-step strategy. Generally, two-dimensional (2D) MoS_2_ nanosheets are firstly manufactured through a "top-down" approach. It is well known that MoS_2_ powder is a quasi-layered material composed of alternating Mo and S layers, which allows for the facile fabrication of few-layer 2D MoS_2_ nanosheets through simple ultrasonication-assisted liquid phase exfoliation [50,51]. Transmission electron microscopy (TEM) image reveals that the prepared MoS_2_ nanosheets possess well-dispersed 2D-layered nanostructure morphology after the exfoliation process (Fig. S1). Additionally, atomic force microscopy (AFM) measurement was used to quantitatively determine the thickness of the exfoliated MoS_2_ nanosheets. As shown in the Fig. S2, the AFM height profile confirms that the majority of the MoS_2_ nanosheets exhibit a thickness of approximately 9.6 nm. Following this, Pt@MoS_2_ composite material was obtained by the in-situ growth of ultrasmall 0D Pt particles on 2D MoS_2_ nanosheets based on spontaneous redox reaction between noble metal precursor H_2_PtCl_6_ and MoS_2_. It has been reported that the work function of liquid-exfoliated MoS_2_ ranges from 5.2 to 5.4 eV, which is significantly higher than the reduction potentials of and PtCl_6_^2−^ [52,53]. During the reaction, the defects and edges on MoS_2_ containing partially unbound sulfur serve as the primary sites for metal core seeding, and the incorporation of precursors stimulates the subsequent growth of Pt NPs. It is noteworthy that existing methods for synthesizing Pt@MoS_2_ hybrids typically require high temperatures, organic solvents, corrosive reagents, and additional reducing agents [[54], [55], [56]]. In contrast, our synthetic approach is greener and milder, proceeding in aqueous phase at lower temperature (60 °C) without requiring extra reducing agents, demonstrating superior cost-effectiveness and greater potential for practical applications.

**Fig. 1:**
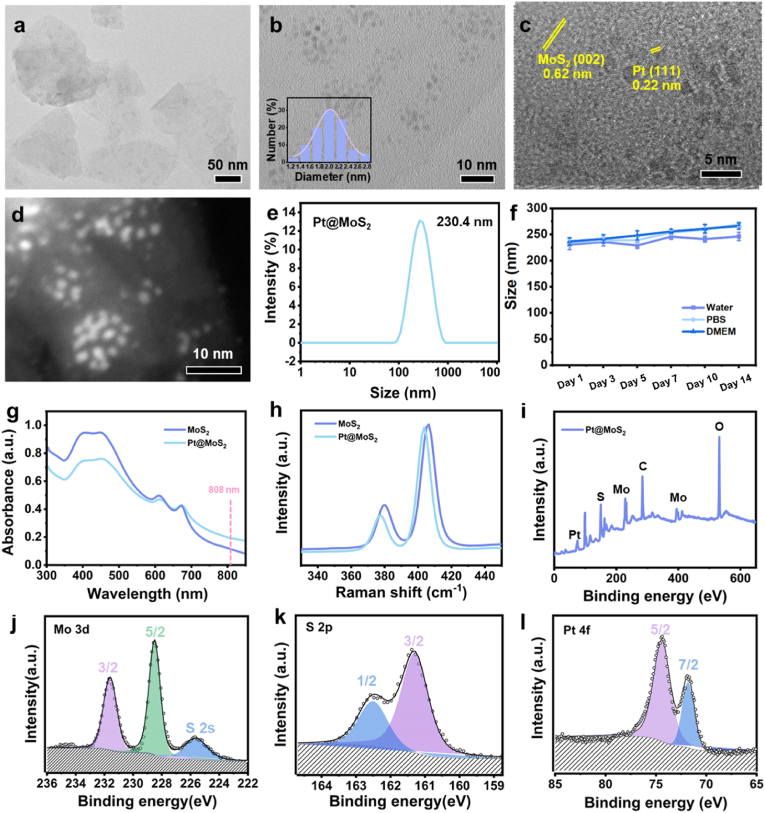
(a) Typical TEM image of Pt@MoS_2_. (b) Statistical analysis of Pt sizes in Pt@MoS_2_ determined by TEM. (c) HRTEM image of Pt@MoS_2_. (d) HAADF-STEM image of Pt@MoS_2_. (e) Hydration particle size statistics of Pt@MoS_2_ determined by DLS. (f) Size stability analysis of Pt@MoS_2_ in 14 days determined by DLS in water, PBS (pH = 6.0), and DMEM. (g) UV–Vis absorbance spectra of MoS_2_ and Pt@MoS_2_. (h) Raman curve of MoS_2_ and Pt@MoS_2_. (i) XPS full spectra of Pt@MoS_2_. High-resolution XPS spectra of (j) Mo 3d (k) S 2p and (l) Pt 4f.

As shown in Table S1, the real mol concentration (mol %) of Pt@MoS_2_ nanozyme is determined to be: Pt: 5.8 %, Mo: 94.2 %, indicating the succussful doping of Pt in MoS_2_. Furthermore, the TEM images of Pt@MoS_2_ show that the composite material maintains a uniform 2D nanosheet morphology (Fig. 1a). Additionally, small-sized Pt NPs are observed on the MoS_2_ nanosheets, with an average size of approximately 2 nm (Fig. 1b). In high-resolution TEM (HRTEM) images, lattice fringes are measured to be 0.22 nm and 0.62 nm, which are assigned to the Pt (111) and MoS_2_ (002) planes, respectively (Fig. 1c). Further, high-angle annular dark field scanning transmission electron microscopy (HAADF-STEM) image validates the small-sized Pt particles dispersed on the 2D MoS_2_ nanosheets (Fig. 1d). As shown in Fig. 1e, dynamic light scattering (DLS) test results further confirm the hydrodynamic diameter of Pt@MoS_2_ to be 230.4 ± 9.2 nm with a polydispersity index (PDI) value of 0.232, consistent with the TEM results. The size of Pt@MoS_2_ is slightly larger than that of MoS_2_ (214.6 ± 11.2 nm) due to the Pt doping (Fig. S3). Besides, the zeta potential values of MoS_2_ and Pt@MoS_2_ are measured to be −38.1 mV and −31.2 mV, respectively, indicating their good colloidal stability (Fig. S4). The slight decrease in the zeta potential of MoS_2_ after Pt decoration can be attributed to the partial coverage of negatively charged edge sites on MoS_2_ nanosheets by Pt NPs. Since sulfur terminals primarily contribute to MoS_2_'s surface charge, their shielding by Pt leads to a reduction in the measured zeta potential. Moreover, the Pt@MoS_2_ composite materials were respectively immersed in deionized water, phosphate-buffered saline (PBS) solution (pH = 6.0), and Dulbecco's modified Eagle medium (DMEM) with 10 % fetal bovine serum (FBS) for different times to evaluate the stablity. As shown in Fig. 1f, Pt@MoS_2_ demonstrates excellent stability over a 14-day evaluation period, which is beneficial for its biomedical application. Furthermore, TEM images of Pt@MoS_2_ after incubation in PBS at 37 °C for extended periods (1, 3, 5, and 7 days) clearly show that the nanozyme maintains its structural integrity without noticeable degradation or aggregation throughout the testing period (Fig. S5), which further supports the robustness of our material for potential biomedical applications.

Subsequently, the ultraviolet-visible (UV-vis) absorption spectra of MoS_2_ and Pt@MoS_2_ are tested to study their optical properties. As revealed in Fig. 1g, both MoS_2_ and Pt@MoS_2_ show characteristic absorption peaks at 610 nm and 670 nm, indicating the presence of layered 2H-MoS_2_ structures in samples. Additionally, due to Pt doping, the absorption of Pt@MoS_2_ in the longer wavelength region (700–850 nm) is significantly higher than that of MoS_2_, which is beneficial for enhancing the photothermal conversion efficiency under 808 nm laser irradation. Raman spectra reveals that the two characteristic peaks of MoS_2_ (382 cm^−1^ for E_2g_ modes and 408 cm^−1^ for A_1g_ modes) in Pt@MoS_2_ NPs shift to the left by ∼6 cm^−1^ compared to MoS_2_, indicating electron transfer from Pt to MoS_2_ in Pt@MoS_2_ heterojunction structure (Fig. 1h). As shown in Fig. S6, the XRD patterns of the MoS_2_ have primary diffraction peak that occurred at 2θ = 13.9° corresponding to (002) plane (JCPDS 00-002-1133). After Pt loading, the Pt@MoS_2_ nanocomposite shows two new distinct peaks at 39.8° and 46.4° corresponding to the (111) and (200) peaks of Pt (JCPDS 87–0646).

After that, X-ray photoelectron spectroscopy (XPS) measurement is performed to reveal the element composition in the nanocomposites. As shown in Fig. 1i, the XPS full spectrum reveal the expected characteristic peaks from Mo, S, and Pt elements in the Pt@MoS_2_ nanozyme. Moreover, the high-resolution Mo 3d spectrum clearly displays characteristic peaks at 228.82 eV and 232.18 eV, corresponding to the 3d_5/2_ and 3d_3/2_ of Mo^4+^ in 2H-MoS_2_ (Fig. 1j) [57]. Additionally, the S 2p_3/2_ peak at 161.45 eV and the S 2p_1/2_ peak at 162.48 eV are observed in the high-resolution S 2p spectrum (Fig. 1k) [58]. As shown in Fig. 1l, the high-resolution Pt 4f spectrum distinctly depicts the Pt 4f_7/2_ peak at 71.95 eV and the Pt 4f_5/2_ peak at 74.62 eV, which are exclusively attributed to metallic Pt(0) without any detectable satellite peaks corresponding to Pt(II) or Pt(IV) species [59]. This unambiguous spectral signature provides direct evidence for the complete reduction of precursors (H_2_PtCl_6_) to metallic Pt(0) during synthesis, confirming the successful doping of Pt NPs in Pt@MoS_2_. Overall, the above experiments demonstrate the successful fabrication of the Pt@MoS_2_ composite material.

### 2.2 Photothermal and nanozyme performance

**Fig. 2:**
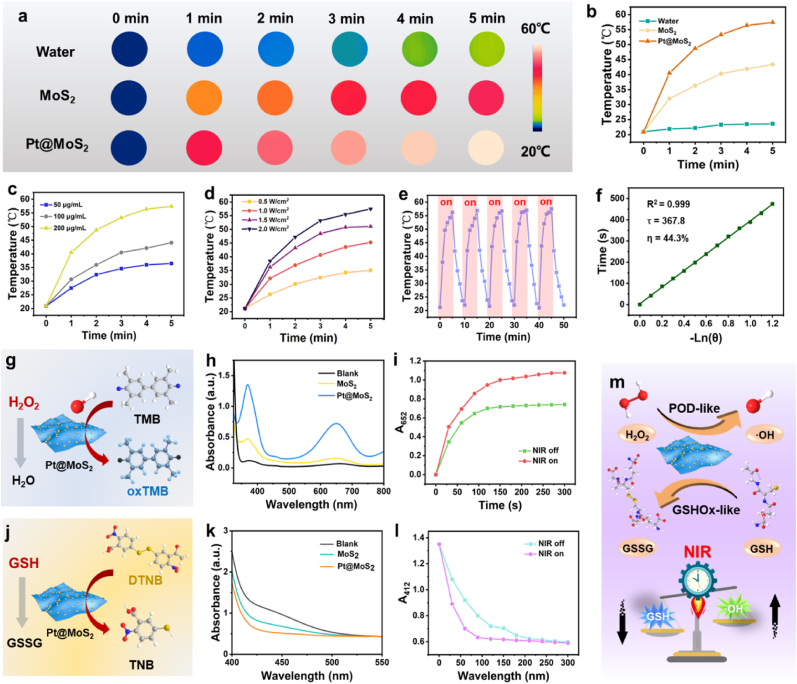
Photothermal images (a) and curves (b) of water, MoS_2_ and Pt@MoS_2_ solutions (200 μg/mL) under 808 nm laser irradation (1.0 W/cm^2^) for 5 min. (c) Photothermal curves of Pt@MoS_2_ at various concentrations under 808 nm laer irradation (1.0 W/cm^2^) for 5 min. (d) Photothermal curves of Pt@MoS_2_ (100 μg/mL) at different power densities. (e) Photothermal curves of Pt@MoS_2_ for five NIR laser on/off cycles. (f) Photothermal conversion efficiency (η) of Pt@MoS_2_. (g) Schematic diagram of TMB assay for testing POD-like catalytic activity of Pt@MoS_2_. (h) UV–Vis absorbance spectra of TMB solution catalyzed with MoS_2_ and Pt@MoS_2_. (i) Absorbance changes at 652 nm of TMB solution catalyzed with Pt@MoS_2_ under NIR laser on/off conditions. (j) Schematic diagram of DTNB assay for testing GSHOx-like catalytic activity of Pt@MoS_2_. (k) UV–Vis absorbance spectra of DTNB solution catalyzed with MoS_2_ and Pt@MoS_2_. (l) Absorbance changes at 412 nm of DTNB solution catalyzed with Pt@MoS_2_ under NIR laser on/off conditions. (m) Schematic diagram of NIR-assisted dual-enzyme activity of Pt@MoS_2_ NCs for modulating TME.

Both Pt and MoS_2_ have been reported to exhibit impressive optical absorption in the NIR region, which inspired us to investigate their photothermal conversion characteristics triggered by NIR laser [60,61]. In this work, we selected the 808 nm laser in photothermal performance study for the following reasons: (1) Compared to short-wavelength lasers such as 600 nm, the 808 nm laser provides sufficient penetration depth to effectively target deep-seated tumors, which aligns with our therapeutic objectives; (2) In contrast to longer-wavelength lasers (e.g., 1064 nm), the 808 nm laser is widely adopted in both clinical and research settings due to its relatively lower cost and compatibility with commonly used equipment; (3) The 808 nm laser matches well with the strong near-infrared absorption characteristics of our Pt@MoS_2_ nanozyme (Fig. 1g), ensuring efficient photothermal conversion. As shown in Fig. 2a and b, compared to MoS_2_, Pt@MoS_2_ at the same dosage demonstrates significantly enhanced photothermal conversion capability with increasing irradiation time of the 808 nm laser. Moreover, as shown in Fig. 2c and d, Pt@MoS_2_ exhibits elevated temperatures with increasing concentration and irradiation power density. Notably, Pt@MoS_2_ maintains high photothermal stability after five on/off cycles of 808 nm laser irradiation (Fig. 2e). In addition, the calculated photothermal conversion efficiency of Pt@MoS_2_ is 44.3 %, which is significantly higher than that of MoS_2_ (Fig. 2f and S7). The improved photothermal conversion efficiency makes Pt@MoS_2_ ideal candidate for further tumor photothermal therapy.

**Fig. 3:**
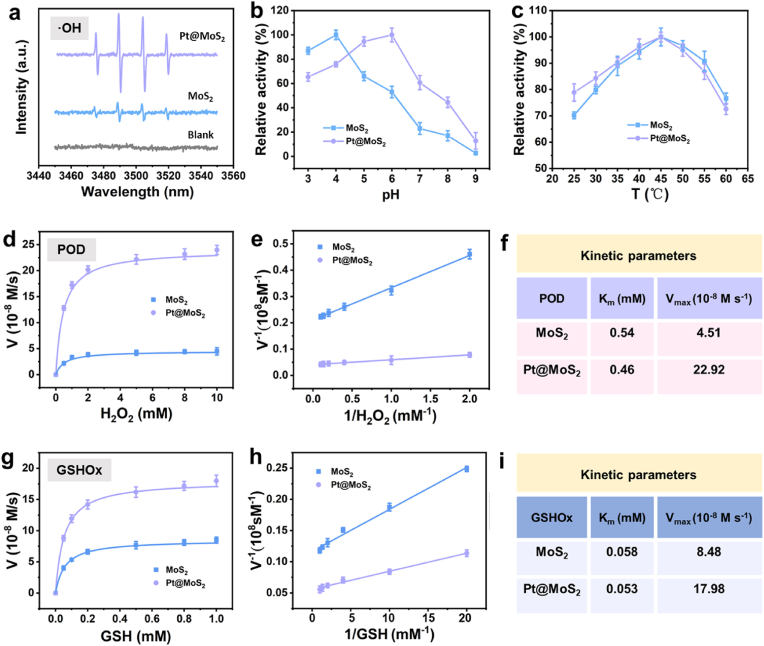
(a) EPR spectra of ·OH formation during the POD-like catalysis of MoS_2_ and Pt@MoS_2_. Relative activity of POD-like activity of MoS_2_ and Pt@MoS_2_ under different pH values (b) and temperatures (c). (d) Michaelis-Menten plots for POD-like activity of MoS_2_ and Pt@MoS_2_. (e) Lineweaver-Burk plots for GSHOx-like activity of MoS_2_ and Pt@MoS_2_. (f) Kinetic parameters for POD-like activity of MoS_2_ and Pt@MoS_2_. (g) Michaelis-Menten plots for POD-like activity of MoS_2_ and Pt@MoS_2_. (h) Lineweaver-Burk plots for GSHOx-like activity of MoS_2_ and Pt@MoS_2_. (i) Kinetic parameters for GSHOx-like activity of MoS_2_ and Pt@MoS_2_.

The utilization of multi-enzyme-like catalytic activities in nanozymes has been established as an emerging strategy for precision tumor therapy [62,63]. Previous studies have demonstrated that bimetallic nanozymes exhibit superior performance in tumor catalytic therapy, attributed to the enhanced flexibility of catalytic sites between dual active centers [64,65]. Owing to the significance of POD and GSHOx enzyme for redox homeostasis disruption, the two kinds of nanozyme activities of bimetallic Pt@MoS_2_ are then investigated through classic colorimetric reactions. As shown in Fig. 2g, 3,3′,5,5′-tetramethylbenzidine (TMB) is used as the colorimetric substrate to detect the POD-like enzyme activity of the material. In the presence of H_2_O_2_, TMB can be oxidized by the nanozyme to form an oxidation TMB product with a maximum absorption peak at 650 nm. As shown in Fig. 2h, both MoS_2_ and Pt@MoS_2_ possess POD-like catalytic activity, but the relative activity of the MoS_2_ nanozyme is significantly enhanced after Pt doping. Importantly, by monitoring the absorbance of oxidized TMB at 650 nm, the catalytic ability of Pt@MoS_2_ further is increased after NIR laser irradiation, which is consistent with observations in other heterojunction photothermal nanozyme studies (Fig. 2i). To study the GSHOx-like activity of the material, 5,5′-dithiobis-(2-nitrobenzoic acid) (DTNB) is selected as the colorimetric substrate, which can be converted into 5′-thio-2-nitrobenzoic acid (TNB) by the nanozyme in the presence of GSH (Fig. 2j). Therefore, the maximum absorption peak signal of DTNB at 412 nm will be decreased. Fig. 2k indicates that both MoS_2_ and Pt@MoS_2_ possess GSHOx-like activity, but the GSHOx activity of MoS_2_ is significantly enhanced after Pt doping. Importantly, by monitoring the absorbance of DTNB at 412 nm, the catalytic ability of Pt@MoS_2_ is further increased after NIR laser irradiation, which is similar to POD activity (Fig. 2l). The significantly enhanced dual-enzyme activity may be attributed to the synergistic coupling at the 0D/2D heterojunction of Pt@MoS_2_. Additionally, the photothermal effect of the material leads to an increase in the reaction system temperature, which is beneficial for the transfer of electrons at the heterojunction interface, thereby accelerating the catalytic reaction. Our multifunctional nanozymes can effectively target the characteristics of ROS and GSH in the TME, break the oxidative stress balance, and combine with photothermal therapy to amplify the antitumor effect (Fig. 2m). Overall, the developed nanozyme with excellent photothermal, POD-like, and GSHOx-like activities is expected to have great application potential in the field of tumor treatment.

To elucidate the potential mechanism of Pt noble metal doping on the nanozyme activity of single-metal MoS_2_, the catalytic activity of the nanozyme is further investigated. As shown in Fig. 3a, electron paramagnetic resonance (EPR) spectra strongly demonstrate that more hydroxyl radicals are generated during the POD-like catalytic process of Pt@MoS_2_ in a weakly acidic environment (pH = 6.0). Furthermore, the formation of the Pt@MoS_2_ composite material results in a significant shift in the optimal pH for maximum enzyme activity from 4.0 for single-metal MoS_2_ to 6.0, indicating that Pt doping can more effectively modulate the POD-like activity of MoS_2_ for better aligning with the characteristics of the TME (Fig. 3b). Fig. 3c shows the influence of temperature on the POD-like enzyme activity of the material. The results indicate that both materials exhibit optimal POD enzyme activity at 45–50 °C, and further temperature increases may lead to a loss of enzyme activity. Moreover, the reactive activity is nearly unchanged during 30 days of storage time at room temperature, indicating its robust catalytic performance (Fig. S8).

Subsequently, the POD-like enzyme kinetics of the two materials were analyzed using the typical Michaelis-Menten equation to obtain the Michaelis constant (K_m_) and maximum reaction rate (V_max_). As shown in Fig. 3d–f, that Pt@MoS_2_ has a lower K_m_ value compared to MoS_2_, indicating a better affinity for the H_2_O_2_ substrate. Simultaneously, the V_max_ value of Pt@MoS_2_ is 5.08 times greater than that of MoS_2_, providing a clear indication of the superior POD-like catalytic activity of Pt@MoS_2_. Additionally, the Michaelis kinetics parameters of Pt@MoS_2_ are examined in the catalysis of GSH oxidation. As illustrated in Fig. 3g-3i, Pt@MoS_2_ demonstrates a higher affinity and catalytic reaction rate for GSH. These findings suggest that Pt doping is an effective strategy for enhancing the catalytic activity of MoS_2_ nanozymes. Besides, compared to other recently reported nanozymes, Pt@MoS_2_ demonstrates remarkably low K_m_ values and high V_max_ values for both H_2_O_2_ and GSH substrates (Tables S2 and S3), indicating that the Pt@MoS_2_ nanozyme exhibits superior catalytic efficiency. These comparisons strongly support Pt@MoS_2_ as a highly efficient dual-functional nanozyme candidate for therapeutic applications.

**Fig. 4:**
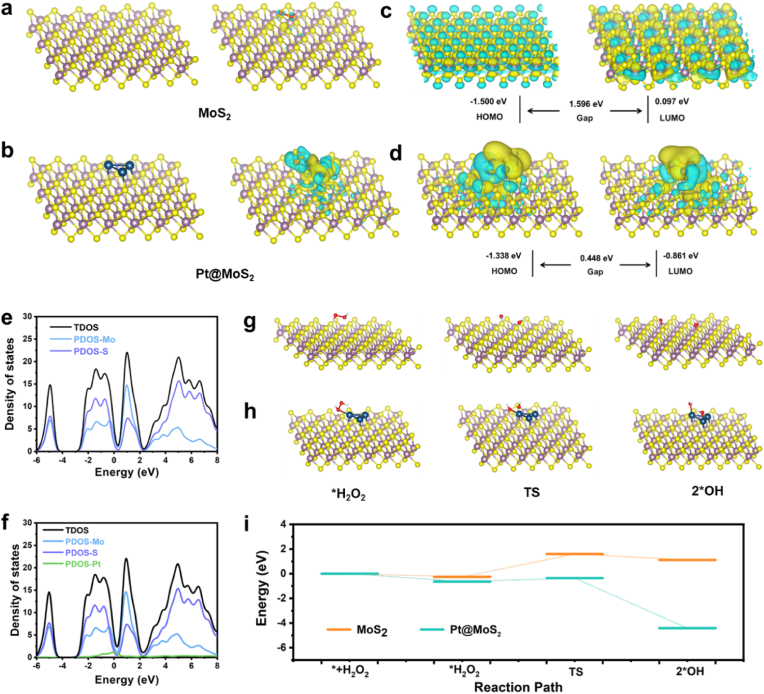
The DFT-optimized configuration and charge density for MoS_2_ (a) and Pt@MoS_2_ (b). The spatial charge distributions of HOMO and LUMO energy level and molecular orbitals for MoS_2_ (c) and Pt@MoS_2_ (d). Density of states curves for MoS_2_ (e) and Pt@MoS_2_ (f). The optimized structures of corresponding reaction intermediates and transition states of H_2_O_2_ adsorption and decomposition on MoS_2_ (g) and Pt@MoS_2_ (h). (i) The energy plot for POD-like catalysis reaction path of MoS_2_ and Pt@MoS_2_.

To further elucidate the potential impact mechanism of Pt doping on the catalytic process, density functional theory (DFT) calculation is employed to interpret the enhancement of catalytic activity of Pt@MoS_2_. Fig. 4a and b displays the fully-optimized geometrical structures of MoS_2_ and Pt@MoS_2_ after DFT optimization, as well as the distribution of electron cloud density upon adsorption of H_2_O_2_ molecules. The results indicate that the electron cloud density at the interface of Pt@MoS_2_ is significantly higher than that of monometallic MoS_2_, suggesting highly active electronic states on the 0D/2D heterointerface. Additionally, the highest occupied molecular orbital (HOMO) and the lowest unoccupied molecular orbital (LUMO) are calculated for both materials. As shown in Fig. 4c and d, the bandgap of Pt@MoS_2_ is 0.448 eV after Pt doping, much lower than that of MoS_2_ at 1.596 eV. These results suggest that there is a higher probability of electron transitions in the Pt@MoS_2_ material, thereby enhancing the efficiency of electron transfer and catalytic capability. Furthermore, the analysis of the density of states (DOS) for MoS_2_ and Pt@MoS_2_ indicates that the bands near the Fermi level are primarily occupied by Mo 4d and S 3p electrons (Fig. 4e). After Pt doping, due to the heterointerface interaction, Pt 4f electrons provide spin polarization, thus affecting the bands near the Fermi level. Moreover, the bandgap analysis demonstrates that there is no significant difference between the minimum conduction band (CBM) and the maximum valence band (VBM) of MoS_2_ and Pt@MoS_2_ (Fig. S9). Compared to MoS_2_, the smaller energy band between the CBM and VBM of Pt@MoS_2_ indicates the enhanced electron-hole separation, which may be the reason for the increased catalytic efficiency. Concurrently, the POD-like catalytic processes of MoS_2_ and Pt@MoS_2_ are simulated based on DFT calculations and the associated energy changes are then computed. As shown in Fig. 4g–i, the POD-like enzyme catalytic process involves the adsorption of H_2_O_2_ on the material surface and the decomposition of H_2_O_2_ into two hydroxyl groups. The calculated adsorption energies of H_2_O_2_ on MoS_2_ and Pt@MoS_2_ are 0.253 eV and 0.632 eV, respectively. indicating that Pt@MoS_2_ The stronger adsorption of Pt@MoS_2_ for H_2_O_2_ is consistent with the experimental results of determined K_m_ values. Additionally, Pt@MoS_2_ has a lower energy barrier for catalyzing the decomposition of H_2_O_2_ into hydroxyl groups and releases more heat. The aforementioned results suggest that the Pt@MoS_2_-mediated catalytic reaction is more favorable than MoS_2_ in both thermodynamics and kinetics. Recent studies have confirmed that the catalytic active sites of MoS_2_ are primarily concentrated on the edges or defect sites [33,66]. Therefore, the catalytic activity of the central majority area of MoS_2_ can be enhanced by Pt doping. We believe that the outstanding nanozyme catalytic capability of Pt@MoS_2_ mainly stems from the formation of the heterointerface.

### 2.3 Cellular ferroptosis therapy performance and mechanism

**Fig. 5:**
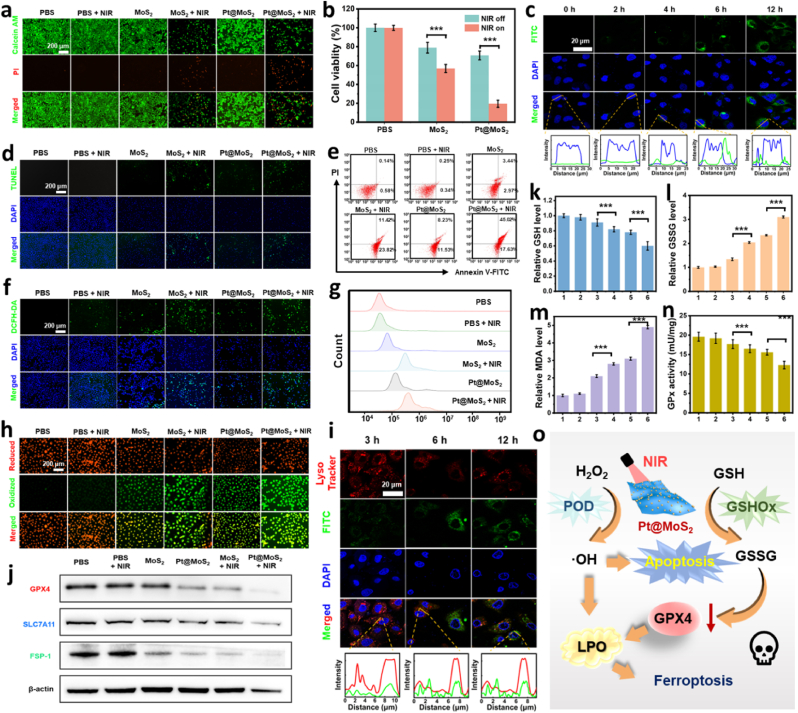
(a) Fluorescence images of Calcein-AM/PI staining after different treatments. (b) Cell viabilities after different treatments. (c) Fluorescence images of cellular uptake of FTIC-labeled Pt@MoS_2_ by HCC cells after different incubation durations. (d) Fluorescence images of TUNEL staining after different treatments. (e) Flow cytometry analysis of apoptosis in different groups after treatment. (f) Fluorescence images of DCFH-DA staining after different treatments. (g) Flow cytometry analysis of intracellular ROS levels in different groups after treatment. (h) Fluorescence images of C11-BODIPY^581/591^ staining after different treatments. (i) Confocal laser scanning microscopy (CLSM) images of the FITC-labeled Pt@MoS_2_ with lysosome after different incubation durations and the corresponding analysis. (j) Western blot for GPX4, SLC7A11, and FSP-1 of HCC cells after different treatments. Relative intracellular GSH content (k), GSSG content (l), MDA levels (m), and GPx activity (n) after different treatments. (o) Schematic diagram of ferroptosis-mediated anticancer mechanism. All NIR laser treatments were performed using an 808 nm laser at 1.0 W/cm^2^ power density for 5 min. Data are expressed as means ± SD (n = 3). ∗p < 0.05, ∗∗p < 0.01, and ∗∗∗p < 0.001.

Inspired by the exciting photothermal performance and dual-enzyme catalytic activity of Pt@MoS_2_, the antitumor activity against HCC *in vitro* is subsequently explored. Here, human hepatoma (Huh7) cell is used as a model cell. First, the cytotoxic effects of different treatments on cancer cells are tested through calcein-AM/propidium iodide (PI) fluorescence staining and CCK-8 assay. As shown in Fig. 5a and b, in the absence of NIR laser irradiation, the death of HCC cells is attributed to the regulation of the TME and catalytic therapy by the nanozyme, thus the *in vitro* antitumor effect of Pt@MoS_2_ is higher than that of MoS_2_. For photothermal treatment alone, the live/dead fluorescence staining images in Fig. 5a showed no red fluorescence from dead cells. Besides, *in vitro* cytotoxicity results confirmed no significant difference before and after PBS + NIR laser irradiation (Fig. 5b), demonstrating that photothermal treatment alone is insufficient to kill cancer cells. However, when NIR laser irradition was combined with Pt@MoS_2_ (200 μg/mL), it enhanced the killing efficiency by 51.2 % compared to material treatment alone, indicating that photothermal therapy plays a crucial role in improving the therapeutic effect of the nanozyme. Moreover, the anticancer effect of Pt@MoS_2_ nanozyme can be further enhanced with increased dosages (Fig. S10). However, the nanozyme show negligible kill effects on normal cells, demonstrating the selective anticancer ability towards TME (Fig. S11).

To further confirm the effective entry of nanozymes into tumor cells and their therapeutic effects, the endocytosis of Pt@MoS_2_ labeled with fluorescein isothiocyanate (FITC) is tracked using confocal microscopy. As shown in Fig. 5c, the green fluorescence in the cytoplasmic region of Huh7 cells gradually intensifies over different time points and reaches its maximum intensity after 12 h during the observation period, demonstrating the time-dependent endocytosis of the nanozyme. Besides, the localization curve indicates that the nanozyme is distributed in the cytoplasm without entry in the nucleus, suggesting that Pt@MoS_2_ will not interfere with the genetic stability of Huh7 cells. Furthermore, TUNEL fluorescence staining confirmed that the Pt@MoS_2_ + NIR therapy can effectively induce cancer cell apoptosis (Fig. 5d). Concurrently, flow cytometry analysis is performed using an Annexin V-FITC apoptosis detection kit. As depicted in Fig. 5e, the Pt@MoS_2_ + NIR group exhibits the highest rate of cell apoptosis (62.65 %), significantly higher than the MoS_2_ + NIR (35.24 %) and Pt@MoS_2_ (19.76 %) groups. The generation of reactive oxygen species (ROS) within cells has been widely proven to be the foundation for inducing cell apoptosis/ferroptosis. Herein, we quantified the intracellular ROS levels using the ROS probe 2′,7′-dichlorofluorescin diacetate (DCFH-DA) through confocal microscopy and flow cytometry. As indicated in Fig. 5f and g, compared to the control group, the other treatment groups show a significant increase in oxidative stress. Among them, the Pt@MoS_2_ + NIR group exhibits the strongest green fluorescence signal, which is attributed to the substantial production of dichlorofluorescein (DCF). The ROS generation is conducive to promoting lipid peroxidation (LPO) and inducing ferroptosis. Subsequently, the Huh7 cells are stained with BODIPY dye, where red fluorescence represented reduced BODIPY and green fluorescence represented oxidized BODIPY. As shown in Fig. 5h, the Pt@MoS_2_ + NIR group exhibits the highest expression of green fluorescence, indicating the most pronounced degree of lipid peroxidation within the cells.

The timely escape of nanomaterials from lysosome is crucial for preventing their premature degradation within cells and enhancing their bioavailability [67,68]. To confirm this, we specifically stained the lysosomes in cells with lysotracker to monitor the entire process. As shown in Fig. 5i, FITC-labeled Pt@MoS_2_ (green) co-localizes with lysosomes (red) after incubation for 3 h, indicating that Pt@MoS_2_ is taken up by lysosomes immediately after entering the cells. However, after 12 h of incubation, a significant separation of the fluorescence co-localization is observed between the Pt@MoS_2_ and lysosomes in the cells after treatment, proving the successful escape of the nanozyme from the lysosomes.

To further decipher the cellular ferroptosis mechanism, the expression of key proteins after treatment with the different groups is evaluated through Western blotting (Fig. 5j). SLC7A11 is an amino acid antiporter located on the cell membrane, which transports cystine into the cell to synthesize GSH [69]. The reduced expression of SLC7A11 leads to decreased GSH synthesis, which in turn inhibits the synthesis of glutathione peroxidase 4 (GPX4), resulting in the loss of lipid peroxide repair capacity [69]. The SLC7A11/GPX4 axis is the main pathway involved in ferroptosis [70]. The Pt@MoS_2_ + NIR group shows the lowest expression of SLC7A11 and GPX4, thereby confirming the effective occurrence of ferroptosis. As another ferroptosis inhibition system, the FSP-1 can protect cells from ferroptosis caused by GPX4 gene deficiency or inhibition [71]. Therefore, the downregulation of FSP-1 content in the Pt@MoS_2_ + NIR group further confirms the occurrence of the ferroptosis pathway. Generally, the reduction of intracellular GSH is a key inducer of GPX4 expression downregulation. As shown in Fig. 5k and l, Pt@MoS_2_ nanozyme with GSHOx mimetic activity can effectively reduce the intracellular GSH content and increase the GSSG content. Moreover, these effects can be further enhanced under NIR laser stimulation, consistent with the *in vitro* catalytic results. High levels of malondialdehyde (MDA) expression reflect the irreversible accumulation of LPO and are proven to be an important marker of tumor cell ferroptosis [18]. As shown in Fig. 5m, the Pt@MoS_2_ nanozyme could induce more MDA production under 808 nm NIR laser irradiation, indirectly proving the excessive expression of LPO. As shown in Fig. 5n, the vitality of GPX4 is also explored using an enzyme-linked immunosorbent assay (ELISA) kit. As expected, the results show that the vitality of GPX4 is significantly reduced after treatment with Pt@MoS_2_ + NIR. The above experimental results fully demonstrate that the novel nano-formulation based on Pt@MoS_2_ can effectively enter cells, escape from lysosomes in a timely manner, exert TME modulation functions and trigger photothermal/catalytic synergistic therapy for inducing ferroptosis. Specifically, the Pt@MoS_2_ nanozyme can effectively increase the ROS levels, reduce the GSH levels, and inhibit GPX4 expression in the TME (Fig. 5o). These behaviors further lead to the overexpression of LPO and the occurrence of apoptosis and ferroptosis in tumor cells for the efficient eradication of cancer cells.

**Fig. 6:**
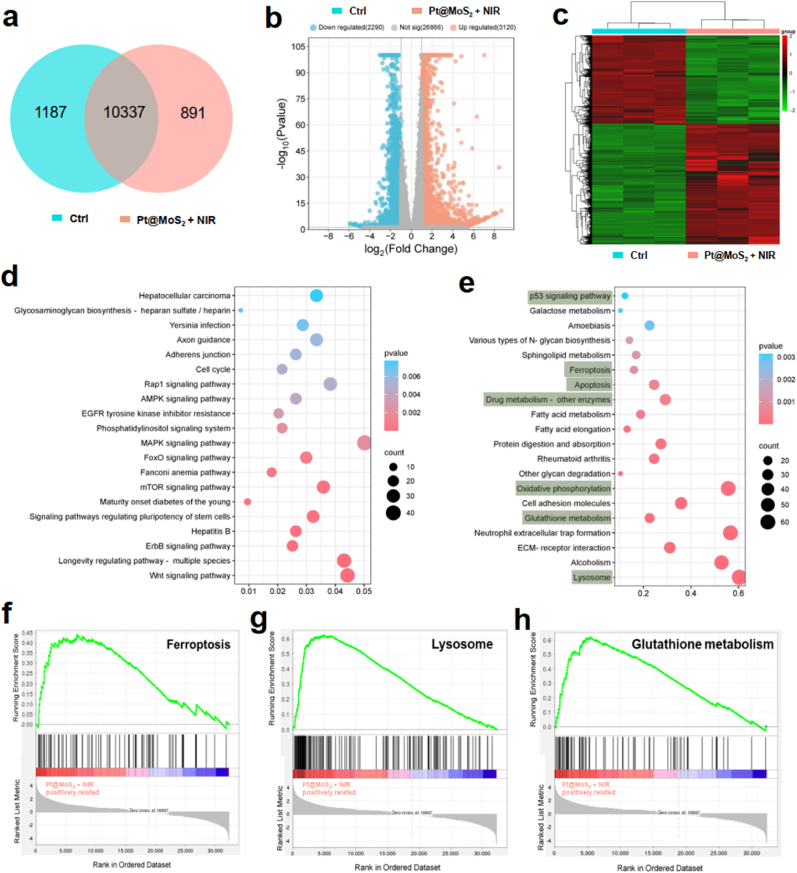
Venn plot (a), Volcano plot (b) and heatmap (c) of gene expression of control and Pt@MoS_2_ + NIR treatment groups after transcriptome sequencing and analysis. Bubble plots of GO enrichment (d) and KEGG enrichment (e) analysis of control and Pt@MoS_2_ + NIR treatment groups. GSEA analysis of ferroptosis (f), lysosome, and glutathione metabolism pathway of control and Pt@MoS_2_ + NIR treatment groups.

Subsequently, to further verify the potential ferroptosis regulatory mechanism of the combined therapy in cancer cells, transcriptome testing and analysis are performed on Pt@MoS_2_ + NIR group and the control group. Firstly, the correlation between the two groups of samples is revealed through Venn diagram distribution analysis. As indicated in Fig. 6a, there are significant differences in the expression genes between the control group and the treatment group. Moreover, Volcano plot-related analysis shows that compared to the control group, Huh7 cells treated with Pt@MoS_2_ + NIR have a total of 3120 upregulated genes and 2290 downregulated genes (Fig. 6b). Additionally, the heat map of related differentially expressed genes indicates that the transcriptome data have high reliability, and the differentially expressed genes can be well clustered and distinguished into different groups (Fig. 6c). Further Kyoto Encyclopedia of Genes and Genomes (KEGG) pathway enrichment analysis shows that the control group exhibits signal pathways closely related to the TME of HCC (Fig. 6d). However, after treatment with Pt@MoS_2_ + NIR, pathways related to cell ferroptosis, apoptosis, oxidative stress, glutathione metabolism, and lysosomes are successfully enriched (Fig. 6e). Moreover, based on gene set enrichment analysis (GSEA), Pt@MoS_2_ + NIR treatment effectively enhanced the signal pathways of ferroptosis, lysosomes, and glutathione metabolism, further confirming the ferroptosis-dependent antitumor mechanism (Fig. 6f–h). The above results offer a deeper understanding of the tumor treatment mechanism of nanozyme and provide valuable insights into the molecular level that the combined therapy can effectively induce ferroptosis in HCC cells.

### 2.4 In vivo anticancer efficacy and biosafety evaluation of Pt@MoS_2_

**Fig. 7:**
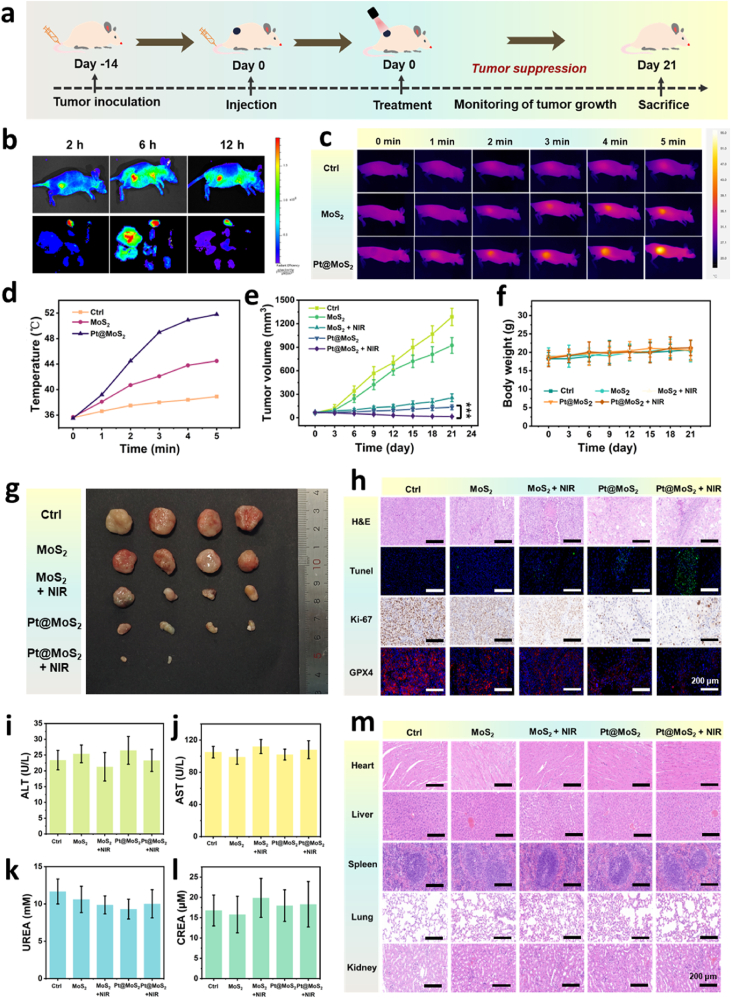
(a) Schematic diagram for the treatment procedure of anti-tumor animal experiments. (b) Fluorescence imaging of mice and organs after intravenous injection with ICG-labeled Pt@MoS_2_ at different time points. Infrared thermal images (c) and temperature curves (d) of tumor region after exposure to 808 nm laser (1.0 W/cm^2^) for 5 min after different treatments. (e) Tumor volume curves of HCC tumor-bearing mice after different treatments in 21 days. (f) Body weight change curves of mice after different treatments in 21 days. (g) Photograph of extracted tumors after different treatments. (h) H&E Tunel, Ki-67, and GPX4 staining analysis of tumor tissue sections after different treatments. Serum levels of ALT (i), AST(j), UREA (k) and CREA (l) of mice after different treatments. (m) H&E images of the major organs of the mice including heart, liver, spleen, lung and kidney after different treatments. Data are expressed as means ± SD (n = 4). ∗p < 0.05, ∗∗p < 0.01, and ∗∗∗p < 0.001.

On the basis of *in vitro* experiments, we further explore the *in vivo* antitumor activity of the developed nanozyme using tumor-bearing BALB/nc mice. Fig. 7a shows the process of the *in vivo* antitumor experiment. Following the establishment of the tumor model, intravenously administered Pt@MoS_2_ effectively accumulated at the tumor site through the enhanced permeability and retention (EPR) effect, thereby exerting therapeutic effects. Subsequently, the bio-distribution of the nanocatalyst is revealed through *in vivo* fluorescence imaging. As depicted in Fig. 7b, Pt@MoS_2_ labeled with fluorescent dyes predominantly accumulated at the tumor site within 2 h post-administration. After 6 h, the distribution of the nanozyme could be observed in the liver organ. After 12 h, only a small amount of fluorescence signal remained in the tumor site, proving that the nanozyme is completely metabolized by the liver.

To further assess the *in vivo* photothermal activity of the material, the temperature change at the tumor site is monitored using an infrared thermal imager following 5 min of NIR laser irradiation. Fig. 7c and d demonstrate that the tumor can be heated more rapidly with Pt@MoS_2_, which is consistent with the results of the *in vitro* photothermal conversion capability experiments. The changes in tumor volume of the mice are recorded over a 21-day treatment period (Fig. 7e). The results indicate that Pt@MoS_2_ can effectively inhibit tumor growth, and the tumor volume is further reduced after NIR laser irradiation. Moreover, there is no significant change in the body weight of the mice during the treatment period (Fig. 7f). As shown in Fig. 7g, the photograph of the tumor-bearing mice after different treatments intuitively reveal that the HCC tumor is almost completely eradicated following Pt@MoS_2_ + NIR treatment, while noticeable tumors remained in the other groups, confirming the feasibility of photothermal/catalytic combined therapy for antitumor treatment *in vivo*.

At the same time, histological analysis of the tumor tissue is also performed after treatment. As shown in Fig. 7h, hematoxylin-eosin (H&E) staining shows that the Pt@MoS_2_ + NIR group has the highest tumor tissue killing effect. Additionally, TUNEL fluorescence staining and Ki-67 immunostaining also verify that the combined therapy has the highest activity in inducing cell apoptosis and inhibiting cell proliferation. Notably, the immunofluorescence staining results of GPX4 in each treatment group reveal that the red fluorescence signal from GPX4 is most significantly downregulated in the Pt@MoS_2_ + NIR group. This result verifies that the excellent antitumor effect of the nanozyme is mediated by the ferroptosis pathway through the downregulation of GPX4. Collectively, Pt@MoS_2_ can achieve efficient NIR photothermal/catalytic synergistic treatment dependent on the ferroptosis pathway *in vivo*.

To further assess the biosafety of the combined therapy, standard blood biochemical indicators are evaluated for each group of treated mice. After 21 days of treatment, parameters such as white blood cells (WBC), red blood cells (RBC), platelets, lymphocytes, mean platelet volume (MPV), and mean corpuscular volume (MCV) are all within normal ranges, with no evident toxicity or adverse reactions observed (Fig. S12). Besides, the blood compatibility of Pt@MoS_2_ was further evaluated. As shown in Fig. S13, Pt@MoS_2_ demonstrates excellent hemocompatibility, with hemolysis ratios remaining below the 5 % safety threshold (2.5 % at therapeutic concentrations up to 200 μg/mL and 4.8 % at 500 μg/mL) when incubated with human red blood cells for 4 h. The excellent blood compatibility can be attributed to the protective PVP coating and optimal surface charge (−31.2 mV), which collectively prevent adverse interactions with blood components. Additionally, key indicators related to liver and kidney function, including aminotransferase (ALT), aspartate aminotransferase (AST), urea nitrogen (UREA), and creatinine (CREA), exhibit no significant abnormalities (Fig. 7i-l). Moreover, the accumulation levels of Mo element in all organs treated with Pt@MoS_2_ + NIR are extremely low, closely resembling the levels of Mo accumulation in the PBS control group (Fig. S14). This indicates that Pt@MoS_2_ can be effectively degraded and excreted from the living body, suggesting its biotoxicity is negligible. Besides, the H&E staining images of the major tissues (heart, liver, spleen, lung, and kidney) of the mice post-treatment further confirmed that the combined therapy do not induce organ toxicity or damage (Fig. 7m). Collectively, these results demonstrate that the combined therapy mediated by the novel Pt@MoS_2_ nanozyme is a safe and efficient antitumor strategy, holding great promise for the precise treatment of clinical HCC tumor.

## 3 Conclusion

In summary, we have judiciously constructed a novel bimetallic Pt@MoS_2_ hybrid nanozyme in the aqueous phase through a simple two-step method. Benefiting from the synergistic action of the bimetallic sites at the heterojunction interface, the composite material exhibits significantly enhanced POD-like and GSHOx-like dual-enzyme catalytic activity compared to the single-metal MoS_2_. DFT calculations further demonstrate the benefits of the bimetallic nanozyme in improving the POD-like catalytic process. Additionally, with the high photothermal conversion efficiency, Pt@MoS_2_ can achieve synergistic NIR photothermal/nano-catalytic therapy. Specifically, Pt@MoS_2_ effectively increases the ROS level and depletes GSH within tumor tissues, amplifying oxidative stress and reshaping the TME by redox homeostasis disruption. Moreover, the combined therapy can effectively downregulate the expression of GPX4, SLC7A11, and FSP-1, promote the accumulation of LPO, and enhance the efficacy of HCC ferroptosis. This ferroptosis amplification strategy based on multifunctional bimetallic nanozymes is simple and effective, potentially overcoming the complexity of existing treatments and providing a beneficial reference for clinical HCC tumor treatment. Moreover, the rational design of Pt@MoS_2_ also provides valuable insights into exploring the structure-activity relationship of bimetallic nanozymes for expanding the biomedical applications of nanozymes. In future, more precise control and functionalization of 0D-2D bimetallic nanozymes are expected to achieve more efficient combination therapies for various diseases.

## 4 Materials and methods

### 4.1 Preparation of MoS_2_ and Pt@MoS_2_

First, 2D MoS_2_ nanosheets were synthesized according to a previously reported top-down procedure [57]. Briefly, 500 mg of MoS_2_ powder (Macklin) was subjected to liquid-phase exfoliation using an ultrasonic probe with 40 mL of 45 % ethanol as the solvent under an ice bath. To increase the exfoliation efficiency and improve the hydrophilicity of the product, PVP (M_w_ = 2000) was added at a mass ratio of 50:1. After ultrasonication at 500 W for 3 h, the mixture was centrifuged (8000 rpm, 10 min) and washed three times with deionized water to remove large MoS_2_ particles and excess PVP, yielding MoS_2_ nanosheets. To synthesize Pt@MoS_2_, 10 mL of MoS_2_ nanosheets (2 mg/mL) was heated to 60 °C in a flask, and 50 μL of 25 mM H_2_PtCl_6_ (Sigma-Aldrich) was rapidly added while stirring, followed by stirring for another 4 h. Subsequently, the solid product was collected after centrifugation (8000 rpm, 10 min) and washing three times with deionized water.

### 4.2 Instruments and characterization

The structure and dispersion of the materials were observed using a TEM (SU8010, HITACHI). The microstructure of the materials was tested on a HRTEM (JEM-2100F, JEOL). The structure of the materials was tested using a HAADF-STEM (FEI Tecnai G20). The size and zeta potential of the NPs were tested using a Nano ZS90-ze zeta potential analyzer (Malvern). The elemental composition of the nanocatalyst was determined using XPS (Thermo Fisher Scientific). All ultraviolet-visible absorption spectra were recorded using a UV-Vis spectrophotometer (Shimadzu UV-2600). Around 0.1 g of the nanomaterial was first digested in an acid solution of H_2_O_2_, HNO_3_, and HClO_4_ in 2:2:1 vol ratio. The element content was determined using ICP-OES (Agilent 5110). EPR spectroscopy (EMX-10/12, Bruker) was used to verify the generation of radicals during the catalytic process. Photothermal images and temperatures were tested using a smartphone-supported thermal imager (FLIR ONE). Cellular fluorescence imaging was tested using a confocal laser scanning microscope (CLSM, Leica TCS SP8). Flow cytometry (FACSCalibur) was used to analyze the signals of dye-labeled cells.

### 4.3 Photothermal activity evaluation

To test the photothermal activity of the materials, 500 μL of water, MoS_2_, or Pt@MoS_2_ was added to a 48-well plate. An 808 nm laser (Beijing Bangshou Technology) was placed vertically 10 cm away from the well plate and irradiated the plate at a certain power density. First, the same concentration (300 μg/mL) of MoS_2_ and Pt@MoS_2_ was analyzed with 1.0 W/cm^2^ laser irradiation for 5 min. Subsequently, the photothermal activity of different material concentrations and photothermal cycle stability were tested under 1.0 W/cm^2^ laser irradiation for 5 min. At the same time, the temperature rise curve was obtained by irradiating 300 ug/mL Pt@MoS_2_ at different power densities of the laser. Additionally, the photothermal conversion efficiency of the material was calculated according to previous reports [36].

### 4.4 Peroxidase-like activity evaluation

In brief, the POD-like activity of the material was detected using a PBS buffer solution system containing TMB (0.2 mM) and H_2_O_2_ (5 mM) (pH = 6.0). First, the absorption spectra of the mixed solution after adding 300 μg/mL of MoS_2_ and Pt@MoS_2_ for 10 min were recorded and compared. At the same time, the intermediate radicals in the reaction process were determined using EPR technology with DMPO as the scavenger. The relative activity of the catalyst was investigated under different pH and temperature conditions. To demonstrate the influence of NIR on POD-like activity, the catalytic effects of the same concentration (300 μg/mL) of MoS_2_ and Pt@MoS_2_ under NIR laser (808 nm, 1.0 W/cm^2^, 5 min) were compared by monitoring the maximum absorbance of oxidized TMB at 650 nm using UV-Vis spectroscopy.

### 4.5 Glutathione oxidase activity evaluation

In brief, the GSHox-like activity of the material was detected using a PBS buffer solution system containing DTNB (100 μg/mL) and GSH (0.25 mM) (pH = 6.0). First, the absorption spectra of the mixed solution after adding 300 μg/mL of MoS_2_ and Pt@MoS_2_ for 10 min were recorded and compared. At the same time, the intermediate radicals in the reaction process were determined using EPR technology with DMPO as the scavenger. To demonstrate the influence of NIR on GSHox-like activity, the catalytic effects of the same concentration (300 μg/mL) of MoS_2_ and Pt@MoS_2_ under NIR laser (808 nm, 1.0 W/cm^2^, 5 min) were compared by monitoring the maximum absorbance of DTNB at 412 nm using UV-Vis spectroscopy.

### 4.6 Catalytic kinetics determination

To test the kinetics of the above two enzyme catalysis, reactions were carried out with different concentrations of H_2_O_2_ ranging from 0 to 10 mM and TMB (0.2 mM), as well as different concentrations of GSH ranging from 0 to 1 mM and DTNB (300 μg/mL). All experiments were conducted in a PBS buffer solution system mimicking the TME environment (pH = 6.0, 37 °C). According to the previously reported procedure, the initial reaction rates (V_0_) of the catalysts at different substrate concentrations were first obtained [47]. Subsequently, the Michaelis-Menten equation was fitted by plotting V_0_ against substrate concentration. Furthermore, the Lineweaver-Burk plot was used to measure the Michaelis constant (K_m_) and reaction rate (V_max_).

### 4.7 Theoretical calculation details

Density functional theory (DFT) calculations were performed using the Vienna Ab Initio Simulation Package (VASP). The projector-augmented wave (PAW) method was employed to describe the interaction between core and valence electrons. The generalized gradient approximation (GGA) in the form of the Perdew-Burke-Ernzerhof (PBE) functional was used to treat electron exchange and correlation effects. The DFT-D3 method was applied for long-range van der Waals interactions. To ensure accuracy, the energy cutoff for the plane wave basis was set to 400 eV, and a Monkhorst-Pack grid of size 3 × 3 × 1 was adopted, with the residual force on each atom being below 0.05 eV/Å.

### 4.8 Cytotoxicity evaluation

The Huh7 and MIHA cells were purchased from Procell and cultured in the culture medium (DMEM, Thermo Fisher Scientific) at 37 °C under an atmosphere of 5 % CO_2_. The cytotoxicity of the different NPs was assessed utilizing the CCK8 assay. Specifically, cells were plated at a density of 5 × 10^3^ cells per well in a 96-well plate and allocated into distinct groups, which were treated with PBS, MoS_2_, and Pt@MoS_2_, respectively. Each treatment group was further divided into subgroups based on whether they received NIR laser irradiation (808 nm, 1.0 W/cm^2^, 5 min) or not. Subsequently, 10 μL of the CCK-8 reagent was introduced into 100 μL of the culture medium and co-incubated for a duration of 2 h. The absorbance at 450 nm was then quantified using a VarioskanTM LUX microplate reader (Thermo, USA). The cell viability of different treatment groups was calculated using the following formula:

$$\text{Viability}\left(\%\right)=\frac{\text{Sample}\;{\text{OD}}_{450}-\text{Blank}\;{\text{OD}}_{450}}{\text{PBS}\;{\text{OD}}_{450}-\text{Blank}{\text{OD}}_{450}}\times 100\%$$

### 4.9 Live/dead fluorescent staining analysis

Huh7 cells were seeded at a density of 5 × 10^5^ cells per well in a 6-well plate and incubated for 24 h. Subsequently, fresh culture medium containing different materials was added. Afterward, the cells were washed, irradiated with an 808 nm laser for 5 min, and then incubated at 37 °C for an additional 4 h, followed by staining with Calcein-AM and PI. The cells were then washed three times with PBS and observed under a fluorescence microscope.

### 4.10 Cellular uptake analysis

Huh7 cells were seeded at a density of 1 × 10^5^ cells per confocal dish (20 mm) and incubated in a 37 °C incubator for 24 h. FITC-labeled (green) Pt@MoS_2_ samples were added and co-incubated for 2, 4, 6, and 12 h. The culture medium was removed, and the cells were washed three times with PBS. After that cells were stained with Hoechst 33342 (blue) for 15 min, washed three times with PBS again, and imaged under a fluorescence microscope to observe cellular uptake.

### 4.11 Apoptosis analysis

The pro-apoptotic properties of different treatments were assessed using flow cytometry. Huh7 cells were cultured in a 6-well plate at a final density of 5 × 10^5^ cells per well. After overnight incubation, cells were subjected to various treatments and thoroughly washed three times with PBS. Then, these cells were digested with trypsin and centrifuged at 800 rpm for 3 min. Following this, the supernatant was discarded, the cells were collected and gently resuspended in PBS. A total of 5 × 10^4^ to 1 × 10^5^ resuspended cells were collected by centrifugation at 400×*g* for 5 min, the supernatant was discarded, and the cells were resuspended in 500 μL of buffer. After that, 5 μL of Annexin V-FITC and 10 μL of PI were added and gently mixed. The mixture was incubated in the dark at room temperature for 10 min, followed by flow cytometric analysis.

### 4.12 ROS generation in cells

To investigate the effects of different treatments on ROS generation in Huh7 cells, the fluorescent probe 2′,7′-dichlorodihydrofluorescein diacetate (DCFH-DA) was used to detect intracellular ROS levels. Cells were firstly cultured in a 96-well plate at a density of 8 × 10^3^ cells per well. After different treatments, the supernatant was discarded and the cells were washed three times with PBS. Next, the DCFH-DA probe was added to the wells and incubated for another 1 h to react with intracellular ROS and generate a fluorescent signal. The cells were then washed with PBS to remove unbound dye. The results were observed under an inverted fluorescence microscope. Additionally, the intracellular ROS levels were detected by flow cytometry.

### 4.13 LPO determination

Following the manufacturer's instructions, cells were seeded at a density of 5 × 10^5^ per well in a 6-well plate and incubated overnight. Afterward, Huh7 cells were treated according to their groups. The culture medium was discarded and the cells were washed with PBS for 3 times. Following this, the cells were incubated with the C11-BODIPY 581/591 fluorescent probe (10 μM) in the dark for 30 min and washed three times with PBS. Finally, cell images were captured under an inverted fluorescence microscope.

### 4.14 Lysosomal escape analysis

Huh7 cells were seeded at a density of 1 × 10^5^ cells in a confocal dish (15 mm) and incubated overnight. Then, FITC-labeled Pt@MoS_2_ nanozyme was co-incubated with the cells for 3, 6, and 12 h. The culture medium was removed and the cells were washed three times with PBS. Lysotracker Red reagent was incubated with the cells at a concentration of 50 nM at 37 °C in the dark for 30 min. After washed three times with PBS, the nuclei were stained with Hoechst 33342 for another 15 min, and the staining results were monitored using CLSM.

### 4.15 Western blot analysis

Proteins were extracted utilizing a nuclear and cytoplasmic protein extraction kit and subsequently stored at −80 °C for future applications. For protein denaturation, solutions derived from differently treated cells were combined with a 1x protein loading buffer in a 4:1 ratio, then denatured in a metallic bath at 100 °C for a period of 10 min, and preserved at −20 °C for subsequent use. For SDS-PAGE electrophoresis, a 10 % stacking gel was employed to resolve the samples within a 10 % SDS-PAGE gel matrix. Electrophoresis was halted once the bromophenol blue dye front migrated approximately 1 cm from the gel's base. The resolved proteins were then transferred onto a polyvinylidene fluoride (PVDF) membrane and blocked with a 5 % solution of skim milk at ambient temperature for 1 h. The membrane was subsequently incubated with the appropriate primary and secondary antibodies, washed with Tris-buffered saline containing Tween 20 (TBST), and stained with enhanced chemiluminescent (ECL) detection reagents. The proteins were visualized using a digital gel imaging system, and their expression levels were semi-quantitatively assessed employing ImageJ software.

### 4.16 MDA concentration, GSH concentration, and GPX4 activity tests

According to the instructions provided by the manufacturer, the levels of GSH, MDA, and GPX activity in cells after various treatments were tested using the GSH, MDA, and GPX activity detection kits (Beyotime). For the determination of GSH and GSSG levels, Huh7 cells were harvested by centrifugation, vortexed thoroughly after the addition of a deproteinizing agent, subjected to two rapid freeze-thaw cycles, and then incubated on ice for 5 min. Subsequently, the cells were centrifuged at 10000 g at 4 °C for 10 min, and the supernatant was collected, diluted, and mixed with a GSH removal auxiliary solution. After vortexing, a GSH removal working solution was added, and the reaction was allowed to proceed at 25 °C for 1 h. The absorbance at 412 nm was measured using a VarioskanTM LUX microplate reader (Thermo, USA), and the concentrations of GSH and GSSG were calculated based on a standard curve. For MDA level detection, cells were lysed on ice using IP cell lysis buffer (P0013), and the protein was quantified using BCA method after lysis. The prepared MDA detection working solution was mixed with samples, standards, and PBS according to the detection reaction system. After heating in boiling water for 15 min and cooling to room temperature, the mixture was centrifuged at 10000 g at room temperature for 10 min, and the absorbance was measured at 532 nm. The MDA content in the samples was calculated according to the standard curve. For GPX4 level detection, cells were lysed on ice using IP cell lysis buffer (P0013), and the supernatant was taken for enzyme activity determination after centrifugation at 10000*g* at low temperature for 10 min. The GPX4 detection working solution was prepared according to the instructions, incubated at room temperature for 15 min, and then 10 μL of peroxide reagent solution was added to each well and mixed. After acting for 5 min, the absorbance at 340 nm was measured, and the GPX4 enzyme activity was calculated according to the standard curve.

### 4.17 Transcriptomic analysis

Initially, total RNA was extracted from cells using TRIzol reagent, and the RNA concentration and purity were determined using NanoDrop, ensuring that the A260/A280 ratio was between 1.8 and 2.1. The mRNA library was constructed using the Illumina TruSeq RNA Sample Preparation Kit. rRNA was first removed from the samples, followed by the capture of mRNA using magnetic beads and reverse transcription to synthesize cDNA, after which the cDNA library was amplified. The quality and fragment size of the library were assessed using the Agilent 2100 Bioanalyzer to ensure its suitability for subsequent sequencing. High-throughput sequencing was performed using the Illumina HiSeq platform. The cleaned sequences were aligned with the reference genome using the HISAT2 alignment tool to obtain the expression levels of each gene. Differential gene expression analysis was conducted using DESeq2 to screen for significantly differentially expressed genes with |log2 fold change| > 2 and p-value <0.05. Gene ontology (GO), Kyoto Encyclopedia of Genes and Genomes (KEGG), and gene set enrichment analysis (GSEA) were performed on the differentially expressed genes, and these steps were completed by Novogene Company.

### 4.18 *In vivo* antitumor and biosafety evaluation

To evaluate the antitumor efficacy of the developed nanozyme, six-week-old BALB/c nude mice were selected as the research model. A total of 10^6^ Huh7 cells were subcutaneously injected into the right forelimb of the nude mice to establish the tumor model. Once the tumor volume reached approximately 50 mm^3^, the tumor-bearing nude mice were randomly divided into five groups (n = 4): (1) control group (PBS group); (2) MoS_2_; (3) MoS_2_ + NIR; (4) Pt@MoS_2_; (5) Pt@MoS_2_ + NIR. All NIR laser treatments were performed using an 808 nm laser at 1.0 W/cm^2^ power density for 5 min. The tumor volume was calculated using the formula: Volume = (length) × (width)^2^/2.

For *in vivo* fluorescence imaging, ICG-labeled Pt@MoS_2_ was intravenously injected into the mice, and imaging was performed at 2, 6, and 12-h post-injection to track the distribution of the NPs. Tumors were irradiated with an 808 nm laser for 10 min, and photothermal images were captured for each group of mice treated with PBS, MoS_2_, and Pt@MoS_2_ using a photothermal imager. The local tumor temperature of each group was recorded for comparison. Tumor volume and mouse body weight were monitored and recorded every 3 days. On day 21, the mice were sacrificed, and major organs (heart, liver, spleen, lung, kidney) along with tumors were harvested for examination. The organs and tumors were stained with H&E for histopathological analysis. Additionally, tumor tissues were examined for the expression of key molecules such as TUNEL, Ki-67, and GPX4 using immunofluorescence staining. The blood compatibility of Pt@MoS_2_ was further evaluated using previously reported assay [72]. After treatment, blood samples were collected in anticoagulant tubes for further hematological analysis. Finally, the levels of ALT, AST, UREA, and CREA in the serum of differently treated BALB/c nude mice were detected to assess the potential systemic toxicity. All animal experiments in this study were conducted in strict accordance with the Guide for the Care and Use of Laboratory Animals and were approved by the Animal Ethics Committee of the People's Hospital of Zhejiang Province.

### 4.19 Statistical analysis

The significance of experimental data was assessed using one-way analysis of variance (ANOVA). The calculated probability (p) compared to the control group was ∗p < 0.05, ∗∗p < 0.01, and ∗∗∗p < 0.001.

## CRediT authorship contribution statement

**Mengmeng Dong:** Writing – original draft, Validation, Software, Resources, Methodology, Data curation. **Yimo Wang:** Writing – review & editing, Writing – original draft, Software, Resources, Methodology, Investigation, Conceptualization. **Lidong Cao:** Writing – review & editing, Resources, Project administration, Investigation, Funding acquisition. **Xiaobin Fei:** Writing – original draft, Methodology, Investigation. **Junjie Qian:** Methodology, Investigation, Formal analysis. **Qingqing Wu:** Writing – original draft, Visualization, Validation, Methodology, Investigation, Formal analysis. **Lu Zhou:** Resources, Methodology, Investigation, Data curation. **Yueqin Zhang:** Resources, Investigation. **Wei Duan:** Writing – review & editing, Visualization, Validation, Supervision, Resources, Formal analysis, Conceptualization. **Chengwu Zhang:** Supervision, Methodology, Investigation, Funding acquisition, Conceptualization. **Changwei Dou:** Writing – review & editing, Visualization, Validation, Supervision, Software, Resources, Project administration, Funding acquisition, Formal analysis, Data curation, Conceptualization.

## Declaration of competing interest

The authors declare that they have no known competing financial interests or personal relationships that could have appeared to influence the work reported in this paper.

## Data Availability

Data will be made available on request.

## References

[1] Yang J.D., Hainaut P., Gores G.J., Amadou A., Plymoth A., Roberts L.R. (2019). A global view of hepatocellular carcinoma: trends, risk, prevention and management. Nat. Rev. Gastroenterol. Hepatol..

[2] Singal A.G., Kanwal F., Llovet J.M. (2023). Global trends in hepatocellular carcinoma epidemiology: implications for screening, prevention and therapy. Nat. Rev. Clin. Oncol..

[3] Fan B., Zhang Y., Zhou L., Xie Z., Liu J., Zhang C., Dou C. (2024). LYRM2 promotes the growth and metastasis of hepatocellular carcinoma via enhancing HIF‐1α‐Dependent glucose metabolic reprogramming. J. Cell Mol. Med..

[4] Gu Z., Wang L., Dong Q., Xu K., Ye J., Shao X., Yang S., Lu C., Chang C., Hou Y. (2023). Aberrant LYZ expression in tumor cells serves as the potential biomarker and target for HCC and promotes tumor progression via csGRP78. Proc. Natl. Acad. Sci..

[5] Greten T.F., Wang X.W., Korangy F. (2015). Current concepts of immune based treatments for patients with HCC: from basic science to novel treatment approaches. Gut.

[6] Villanueva A., Hernandez-Gea V., Llovet J.M. (2013). Medical therapies for hepatocellular carcinoma: a critical view of the evidence. Nat. Rev. Gastroenterol. Hepatol..

[7] Llovet J.M., Pinyol R., Kelley R.K., El-Khoueiry A., Reeves H.L., Wang X.W., Gores G.J., Villanueva A. (2022). Molecular pathogenesis and systemic therapies for hepatocellular carcinoma. Nat. Cancer.

[8] Donne R., Lujambio A. (2023). The liver cancer immune microenvironment: therapeutic implications for hepatocellular carcinoma. Hepatology.

[9] Shi J., Wang Y., Wu Y., Li J., Fu C., Li Y., Xie X., Fan X., Hu Y., Hu C. (2024). Tumor microenvironment ROS/pH cascade-responsive supramolecular nanoplatform with ROS regeneration property for enhanced hepatocellular carcinoma therapy. ACS Appl. Mater. Interfaces.

[10] Xin F., Wu M., Cai Z., Zhang X., Wei Z., Liu X., Liu J. (2021). Tumor microenvironment triggered cascade‐activation nanoplatform for synergistic and precise treatment of hepatocellular carcinoma. Adv. Healthcare Mater..

[11] Niu B., Liao K., Zhou Y., Wen T., Quan G., Pan X., Wu C. (2021). Application of glutathione depletion in cancer therapy: enhanced ROS-based therapy, ferroptosis, and chemotherapy. Biomaterials.

[12] Huang S., Xu Z., Zhi W., Li Y., Hu Y., Zhao F., Zhu X., Miao M., Jia Y. (2024). pH/GSH dual-responsive nanoparticle for auto-amplified tumor therapy of breast cancer. J. Nanobiotechnol..

[13] Xu W., Yang M., Zhang W., Jia W., Zhang H., Zhang Y. (2024). Tumor microenvironment responsive nano-platform for overcoming sorafenib resistance of hepatocellular carcinoma. Mater. Today Bio.

[14] Tang B., Zhu J., Wang Y., Chen W., Fang S., Mao W., Xu Z., Yang Y., Weng Q., Zhao Z. (2023). Targeted xCT‐mediated ferroptosis and protumoral polarization of macrophages is effective against HCC and enhances the efficacy of the anti‐PD‐1/L1 response. Adv. Sci..

[15] Meng J., Yang X., Huang J., Tuo Z., Hu Y., Liao Z., Tian Y., Deng S., Deng Y., Zhou Z. (2023). Ferroptosis‐enhanced immunotherapy with an injectable dextran‐chitosan hydrogel for the treatment of malignant ascites in hepatocellular carcinoma. Adv. Sci..

[16] Li Y., Liu J., Chen Y., Weichselbaum R.R., Lin W. (2024). Nanoparticles synergize ferroptosis and cuproptosis to potentiate cancer immunotherapy. Adv. Sci..

[17] Wu C., Liu Z., Chen Z., Xu D., Chen L., Lin H., Shi J. (2021). A nonferrous ferroptosis-like strategy for antioxidant inhibition–synergized nanocatalytic tumor therapeutics. Sci. Adv..

[18] Li J., Liu J., Zhou Z., Wu R., Chen X., Yu C., Stockwell B., Kroemer G., Kang R., Tang D. (2023). Tumor-specific GPX4 degradation enhances ferroptosis-initiated antitumor immune response in mouse models of pancreatic cancer. Sci. Transl. Med..

[19] Liang C., Zhang X., Yang M., Dong X. (2019). Recent progress in ferroptosis inducers for cancer therapy. Adv. Mater..

[20] Zhang Y., Li L., Li Y., Fei Y., Xue C., Yao X., Zhao Y., Wang X., Li M., Luo Z. (2022). An ROS‐Activatable nanoassembly remodulates tumor cell metabolism for enhanced ferroptosis therapy. Adv. Healthcare Mater..

[21] Wang Z., Wang X., Dai X., Xu T., Qian X., Chang M., Chen Y. (2024). 2D catalytic nanozyme enables Cascade enzyodynamic effect‐boosted and Ca^2+^ overload‐induced synergistic ferroptosis/apoptosis in tumor. Adv. Mater..

[22] Cao C., Yang N., Su Y., Zhang Z., Wang C., Song X., Chen P., Wang W., Dong X. (2022). Starvation, ferroptosis, and prodrug therapy synergistically enabled by a cytochrome c oxidase like nanozyme. Adv. Mater..

[23] Li S., Zhang Y., Wang Q., Lin A., Wei H. (2021). Nanozyme-enabled analytical chemistry. Anal. Chem..

[24] Wang H., Wan K., Shi X. (2019). Recent advances in nanozyme research. Adv. Mater..

[25] Tang G., He J., Liu J., Yan X., Fan K. (2021).

[26] Tian Q., Li S., Tang Z., Zhang Z., Du D., Zhang X., Niu X., Lin Y. (2024). Nanozyme‐enabled biomedical diagnosis: advances, trends, and challenges. Adv. Healthcare Mater..

[27] Ai Y., Hu Z.N., Liang X., Sun H.b., Xin H., Liang Q. (2022). Recent advances in nanozymes: from matters to bioapplications. Adv. Funct. Mater..

[28] Li S., Shang L., Xu B., Wang S., Gu K., Wu Q., Sun Y., Zhang Q., Yang H., Zhang F. (2019). A nanozyme with photo‐enhanced dual enzyme‐like activities for deep pancreatic cancer therapy. Angew. Chem..

[29] Xing Y., Yasinjan F., Sun S., Yang J., Du Y., Zhang H., Liang Y., Geng H., Wang Y., Sun J. (2024). Nanozyme-based cancer theranostics: a scientometric analysis and comprehensive review. Nano Today.

[30] Zhang X., Chen X., Zhao Y. (2022). Nanozymes: versatile platforms for cancer diagnosis and therapy. Nano-Micro Lett..

[31] Qin S., Zhao H.-y., Luo X.-y., Wang F., Liu J., Ding Y., Hu Y. (2024). Photothermally reinforced nanozyme remodeling tumor microenvironment of redox and metabolic homeostasis to enhance ferroptosis in tumor therapy. ACS Nano.

[32] Zhang Y., Yu W., Chen M., Zhang B., Zhang L., Li P. (2023). The applications of nanozymes in cancer therapy: based on regulating pyroptosis, ferroptosis and autophagy of tumor cells. Nanoscale.

[33] Wang L., Zhang X., You Z., Yang Z., Guo M., Guo J., Liu H., Zhang X., Wang Z., Wang A. (2023). A molybdenum disulfide nanozyme with charge‐enhanced activity for ultrasound‐mediated cascade‐catalytic tumor ferroptosis. Angew. Chem. Int. Ed..

[34] Ding S.-s., He L., Bian X.-w., Tian G. (2020). Metal-organic frameworks-based nanozymes for combined cancer therapy. Nano Today.

[35] Liu Q., Chen Q., Deng X., Zhu Y., Shi Z., Miao R., Ma M., Ran N., Li C., Chen H. (2024). Fe/Co bimetallic nanozyme disrupting tumorous self-regulatory pathways to potentiate ferroptosis. Chem. Eng. J..

[36] Zhao J., Duan W., Liu X., Xi F., Wu J. (2023). Microneedle patch integrated with porous silicon confined dual nanozymes for synergistic and hyperthermia‐enhanced nanocatalytic ferroptosis treatment of melanoma. Adv. Funct. Mater..

[37] Chen C.-C., Dai L., Ma L., Guo R.-T. (2020). Enzymatic degradation of plant biomass and synthetic polymers. Nat. Rev. Chem.

[38] Zhang R., Yan X., Fan K. (2021). Nanozymes inspired by natural enzymes. Acc. Mater. Res..

[39] Wu Y., Zhong H., Xu W., Su R., Qin Y., Qiu Y., Zheng L., Gu W., Hu L., Lv F. (2024). Harmonizing enzyme‐like cofactors to boost nanozyme catalysis. Angew. Chem..

[40] Sheng J., Wu Y., Ding H., Feng K., Shen Y., Zhang Y., Gu N. (2024). Multienzyme‐like nanozymes: regulation, rational design, and application. Adv. Mater..

[41] Pietrzak M., Ivanova P. (2021). Bimetallic and multimetallic nanoparticles as nanozymes. Sensor. Actuator. B Chem..

[42] Li F., Sun H., Ren J., Zhang B., Hu X., Fang C., Lee J., Gu H., Ling D. (2022). A nuclease-mimetic platinum nanozyme induces concurrent DNA platination and oxidative cleavage to overcome cancer drug resistance. Nat. Commun..

[43] Chen Y., Wang P., Hao H., Hong J., Li H., Ji S., Li A., Gao R., Dong J., Han X. (2021). Thermal atomization of platinum nanoparticles into single atoms: an effective strategy for engineering high-performance nanozymes. J. Am. Chem. Soc..

[44] Cursi L., Mirra G., Boselli L., Pompa P.P. (2024). Metrology of platinum nanozymes: mechanistic insights and analytical issues. Adv. Funct. Mater..

[45] Li S., Xu B., Yang H., Zhang C., Chen J., Liu S., Huang Z., Liu H. (2024). A Pt1Pd single‐atom alloy nanozyme with boosted enzyme‐like activity for efficient photo‐mediated tumor therapy. Small.

[46] Ye T., Chen C., Wang D., Huang C., Yan Z., Chen Y., Jin X., Wang X., Ding X., Shen C. (2024). Protective effects of Pt-NC single-atom nanozymes against myocardial ischemia-reperfusion injury. Nat. Commun..

[47] Zhu Y., Zhao R., Feng L., Wang C., Dong S., Zyuzin M.V., Timin A., Hu N., Liu B., Yang P. (2023). Dual nanozyme-driven PtSn bimetallic nanoclusters for metal-enhanced tumor photothermal and catalytic therapy. ACS Nano.

[48] Wu Y.-Y., Tian X., Jiang Y., Ma H.-Y., Wang W., Zhang W.-S., San Martin J., Yan Y., Qin D.-D., Han D.-X. (2024). Advances in bimetallic materials and bimetallic oxide nanozymes: synthesis, classification, catalytic mechanism and application in analytical chemistry. TrAC, Trends Anal. Chem..

[49] Mu S., Yang Y., Han T., Liu J., Zhu Z., Zheng H., Zhang H. (2025). Synergistic surface ligand modification of Ni-Pt bimetallic nanozymes: enhanced catalytic activity and versatile detection of penicillin. Sensor. Actuator. B Chem..

[50] Sethulekshmi A., Saritha A., Joseph K., Aprem A.S., Sisupal S.B. (2022). MoS_2_ based nanomaterials: advanced antibacterial agents for future. J. Contr. Release.

[51] Yu X., Xu C., Sun J., Xu H., Huang H., Gan Z., George A., Ouyang S., Liu F. (2024). Recent developments in two-dimensional molybdenum disulfide-based multimodal cancer theranostics. J. Nanobiotechnol..

[52] Hou H.L., Anichini C., Samorì P., Criado A., Prato M. (2022). 2D Van der Waals heterostructures for chemical sensing. Adv. Funct. Mater..

[53] Liu X., Chen Z., Wang T., Jiang X., Qu X., Duan W., Xi F., He Z., Wu J. (2022). Tissue imprinting on 2D nanoflakes-capped silicon nanowires for lipidomic mass spectrometry imaging and cancer diagnosis. ACS Nano.

[54] Li Y., Wang S., Hu Y., Zhou X., Zhang M., Jia X., Yang Y., Lin B.-L., Chen G. (2022). Highly dispersed Pt nanoparticles on 2D MoS_2_ nanosheets for efficient and stable hydrogen evolution reaction. J. Mater. Chem. A.

[55] He J., Du M., Makvandi P., Xu Y., He X., Jin X. (2025). NIR-II-enhanced Pt-MoS_2_ nanozymes for synergistic photothermal and chemodynamic therapy against methicillin-resistant Staphylococcus aureus to promote wound healing. Nano Res..

[56] Wang J., Cao X., Fang L., You X., Wong K., Cao S., Xiao C., Cai S., Huang Y., Zhang X. (2019). MoS_2_ nanoflower supported Pt nanoparticle as an efficient electrocatalyst for ethanol oxidation reaction. Int. J. Hydrogen Energy.

[57] Li Y., Liu Y., Zhang Y., Dong M., Cao L., Jiang K. (2024). A simple Ag–MoS_2_ hybrid nanozyme-based sensor array for colorimetric identification of biothiols and cancer cells. RSC advances.

[58] Patil S.H., Anothumakkool B., Sathaye S.D., Patil K.R. (2015). Architecturally designed Pt–MoS 2 and Pt–graphene composites for electrocatalytic methanol oxidation. Phys. Chem. Chem. Phys..

[59] Shi Z., Zhang X., Lin X., Liu G., Ling C., Xi S., Chen B., Ge Y., Tan C., Lai Z. (2023). Phase-dependent growth of Pt on MoS_2_ for highly efficient H_2_ evolution. Nature.

[60] Liu S., Pan X., Liu H. (2020). Two‐dimensional nanomaterials for photothermal therapy. Angew. Chem..

[61] Zhi D., Yang T., O'hagan J., Zhang S., Donnelly R.F. (2020). Photothermal therapy. J. Contr. Release.

[62] Ye J., Lv W., Li C., Liu S., Yang X., Zhang J., Wang C., Xu J., Jin G., Li B. (2022). Tumor response and NIR‐II photonic thermal co‐enhanced catalytic therapy based on single‐atom manganese nanozyme. Adv. Funct. Mater..

[63] Liu M., Ye J., Liu S., Xu X., Cui Y., Qu J., Zhang Z., Zhang K., Niu N., Chen L. (2023). Turning silica into enzymes by hydrogenation: simultaneously achieving oxygen vacancy engineering and tumor adaptive accumulation for NIR‐II‐Potentiated therapy. Adv. Funct. Mater..

[64] Ye J., Li C., Xu J., Liu S., Qu J., Wang Q., Cao J., Zhao Y., Li C., Yang P. (2025).

[65] Liu S., Sun Y., Ye J., Li C., Wang Q., Liu M., Cui Y., Wang C., Jin G., Fu Y. (2024). Targeted delivery of active sites by oxygen vacancy-engineered bimetal silicate nanozymes for intratumoral aggregation-potentiated catalytic therapy. ACS Nano.

[66] Feng L., Zhang L., Zhang S., Chen X., Li P., Gao Y., Xie S., Zhang A., Wang H. (2020). Plasma-assisted controllable doping of nitrogen into MoS_2_ nanosheets as efficient nanozymes with enhanced peroxidase-like catalysis activity. ACS Appl. Mater. Interfaces.

[67] Zhao H.-Y., Chen Y.-Q., Luo X.-Y., Cai M.-J., Li J.-Y., Lin X.-Y., Zhang H., Ding H.-M., Jiang G.-L., Hu Y. (2024). Ligand phase Separation-Promoted,“Squeezing-Out” mode explaining the mechanism and implications of neutral nanoparticles that escaped from lysosomes. ACS Nano.

[68] Paillard A., Hindré F., Vignes-Colombeix C., Benoit J.-P., Garcion E. (2010). The importance of endo-lysosomal escape with lipid nanocapsules for drug subcellular bioavailability. Biomaterials.

[69] Su T., Wu G., Zhou P., Wang J., Zhu X., Fan L., Yan H., Ma G., Liu Z., Wang X. (2024). “Resource-Conserving” engineered nanoparticles mediate disulfidptosis by overcoming resistance to ferroptosis for antitumor immunotherapy. Chem. Eng. J..

[70] Zhuang J., Fan R., Liao W., Lin R., Deng A., Zhao T., Hai Y., Li H., Tang L., Wei G. (2024). Organelle synergy unleashed: modulating mitochondrial-endoplasmic reticulum contacts with a self-assembled prodrug amplifies ferroptosis for innovative cancer therapy. Chem. Eng. J..

[71] Guo W., Chen Z., Li Z., Huang H., Ren Y., Li Z., Zhao B., Li G., Hu Y. (2023). Cancer cell membrane biomimetic mesoporous silica nanotheranostics for enhanced Ferroptosis-mediated immuogenic cell death on gastric cancer. Chem. Eng. J..

[72] Duan W., Jin Y., Cui Y., Xi F., Liu X., Wo F., Wu J. (2021). A co-delivery platform for synergistic promotion of angiogenesis based on biodegradable, therapeutic and self-reporting luminescent porous silicon microparticles. Biomaterials.

