# Supplementary material for: Dual enzyme-driven redox homeostasis disruption with ultrasmall Pt-decorated MoS2 for combined ferroptosis therapy of hepatocellular carcinoma
Source: Mater Today Bio. 2025 Aug 30;34:102260. doi: 10.1016/j.mtbio.2025.102260 (PMC12441709; doi:10.1016/j.mtbio.2025.102260)
Supplement: Multimedia component 1 [file mmc1.docx]

Supporting Information

Dual Enzyme-Driven Redox Homeostasis Disruption with Ultrasmall Pt-Decorated MoS_2_ for Combined Ferroptosis Therapy of Hepatocellular Carcinoma

Mengmeng Dong^2^, Yimo Wang^4^, Lidong Cao^1*^, Xiaobin Fei^1^, Junjie Qian^1^, Qingqing Wu^3^, Lu Zhou^1^, Yueqin Zhang^1^, Wei Duan^5*^, Chengwu Zhang^1*^, Changwei Dou^1*^

^1^ General Surgery, Cancer Center, Department of Hepatobiliary & Pancreatic Surgery and Minimally Invasive Surgery, Zhejiang Provincial People’s Hospital, Affiliated People’s Hospital, Hangzhou Medical College, Hangzhou, Zhejiang, China

^2^ Clinical Research Institute, Zhejiang Provincial People's Hospital, Affiliated People’s Hospital, Hangzhou Medical College, Hangzhou, Zhejiang, China

^3^ Department of Surgery, Hangzhou Normal University Second Clinical Medical College, Zhejiang Provincial People's Hospital, Hangzhou, Zhejiang, China

^4^ Department of Radiation Oncology, Tohoky University, Sendai, Japan

^5^ School of Pharmacy, Hangzhou Normal University, Hangzhou, Zhejiang, China

Corresponding Authors: [douchangwei168@sina.com](mailto:douchangwei168@sina.com); [ilkwenxu2019@sina.com](mailto:ilkwenxu2019@sina.com); [duanweichem@163.com](mailto:duanweichem@163.com); [zcwzry@163.com](mailto:zcwzry@163.com).


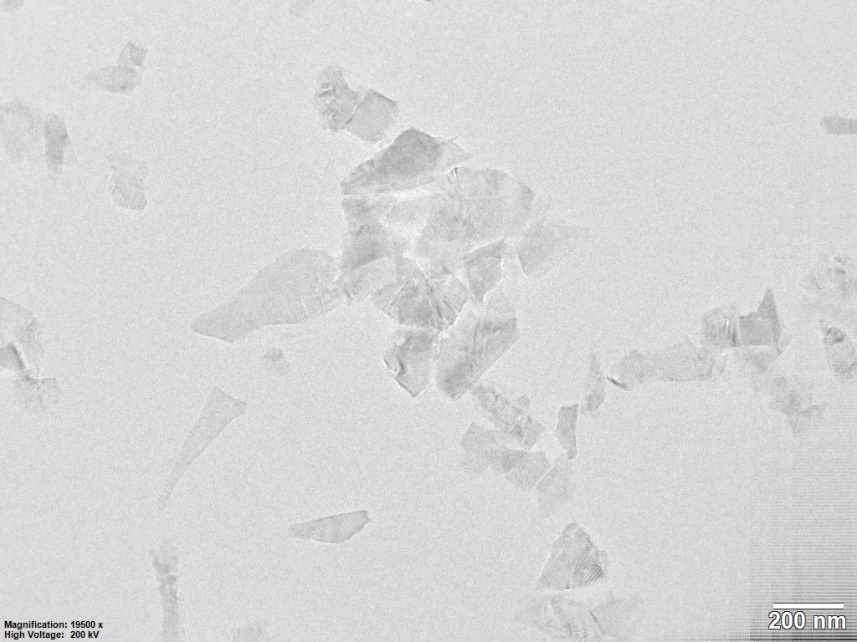


**Figure S1.** TEM image of MoS_2_ nanosheet.


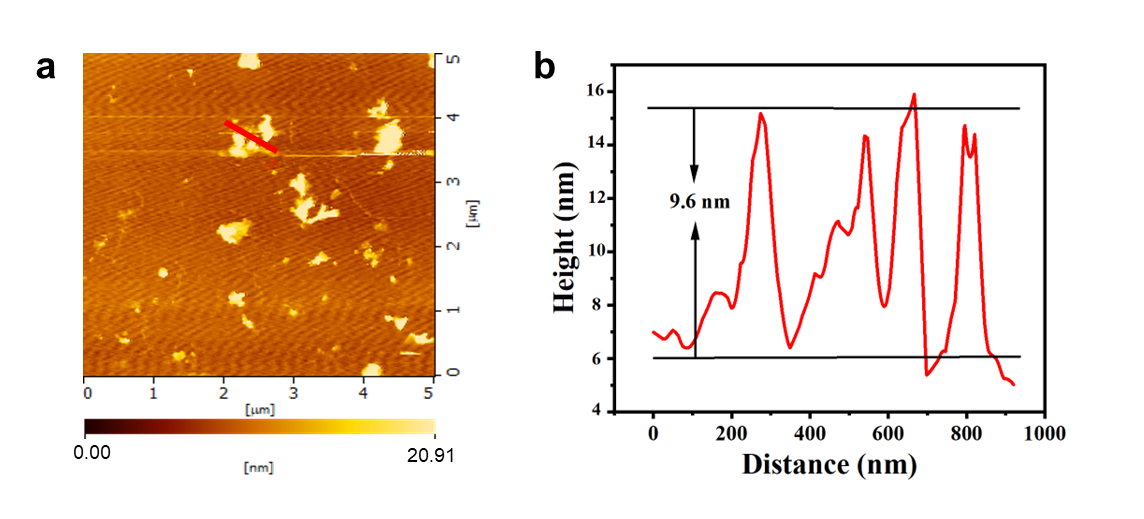


**Figure S2.** (a) AFM image and (b) height profile of MoS_2_ nanosheet.


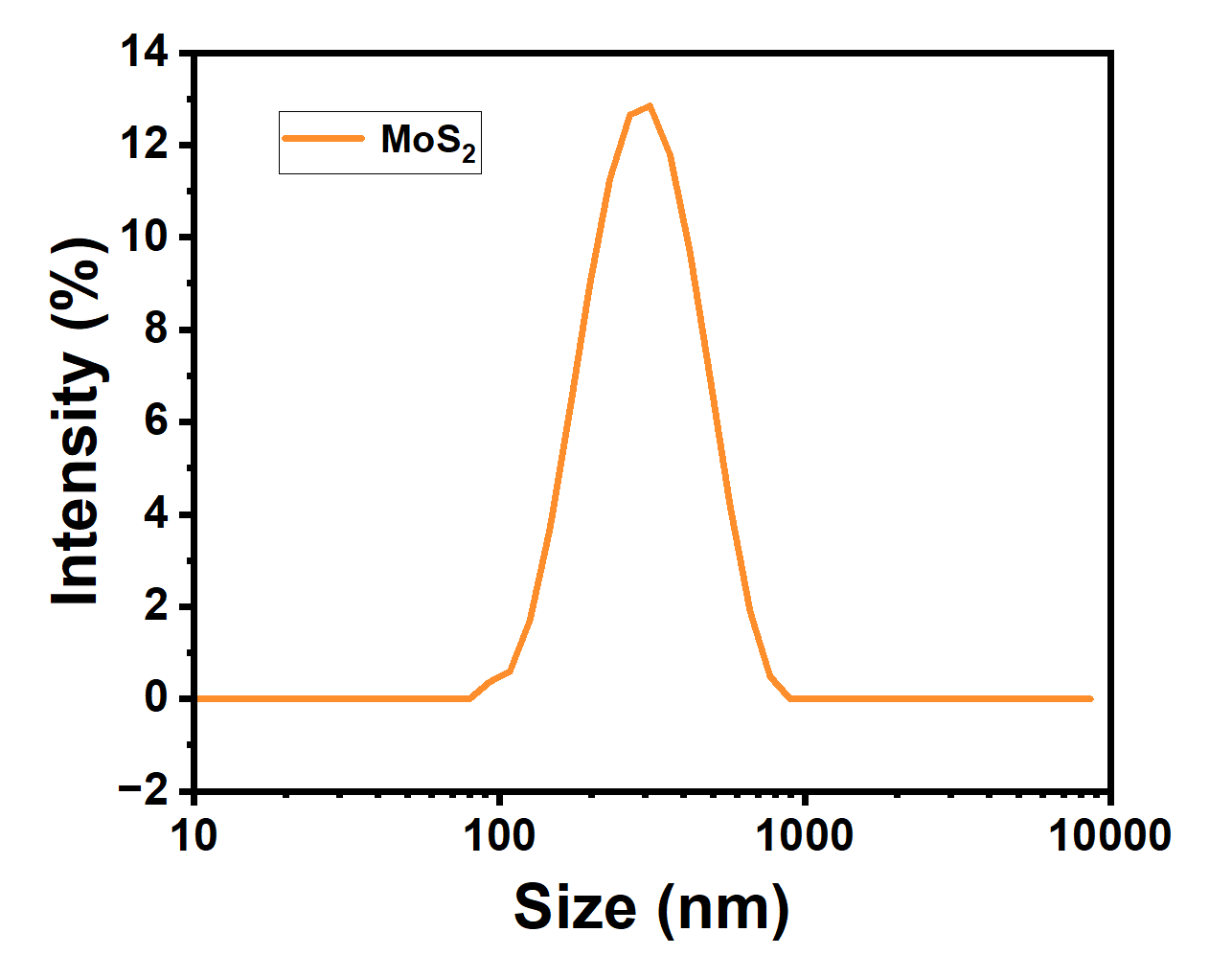


**Figure S3.** DLS test of MoS_2_ nanosheet.


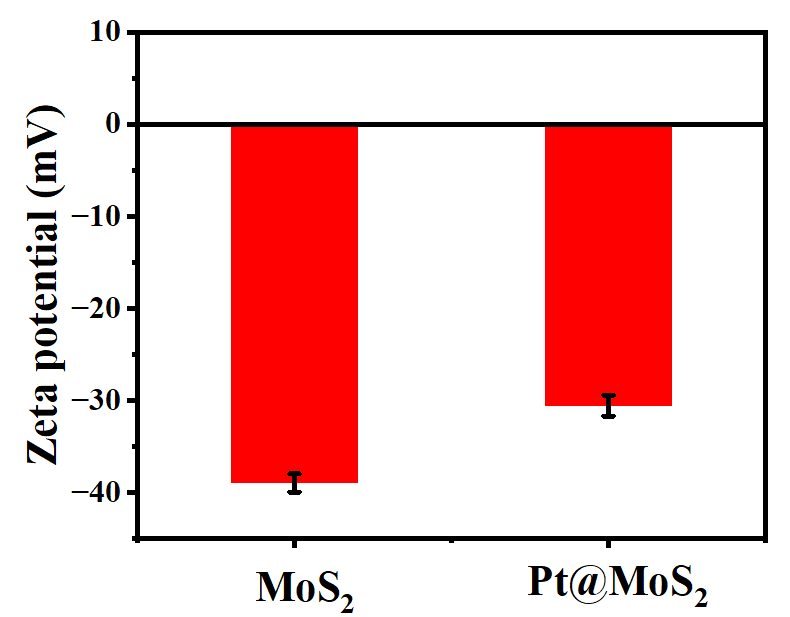


**Figure S4.** Zeta potential of MoS_2_ and Pt@MoS_2_.


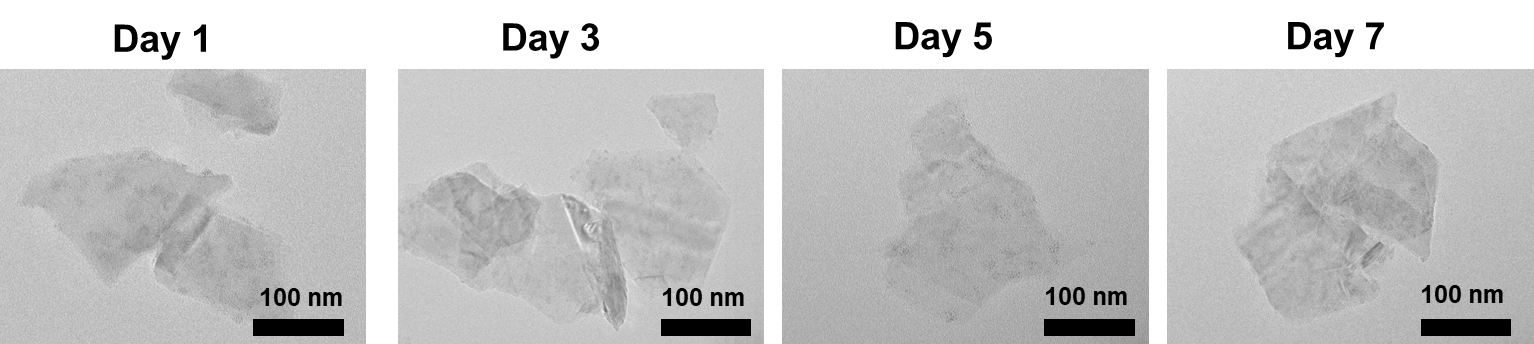


**Figure S5.** TEM image of Pt@MoS_2_ after incubation in PBS at 37°C for extended periods (1, 3, 5, and 7 days)


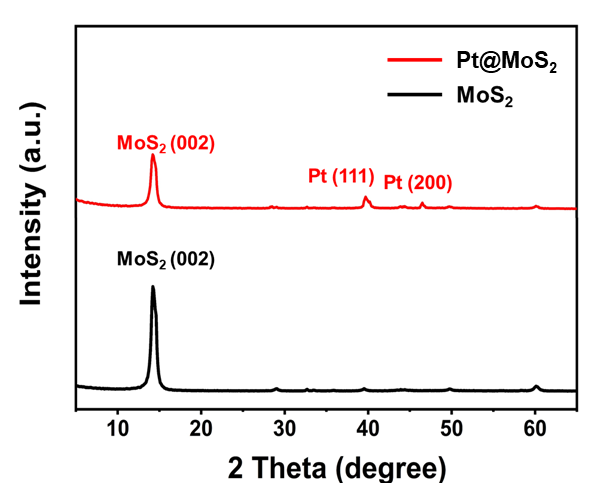


**Figure S6.** XRD patterns of exfoliated MoS_2_ nanosheets and Pt@MoS_2_ hybrids.


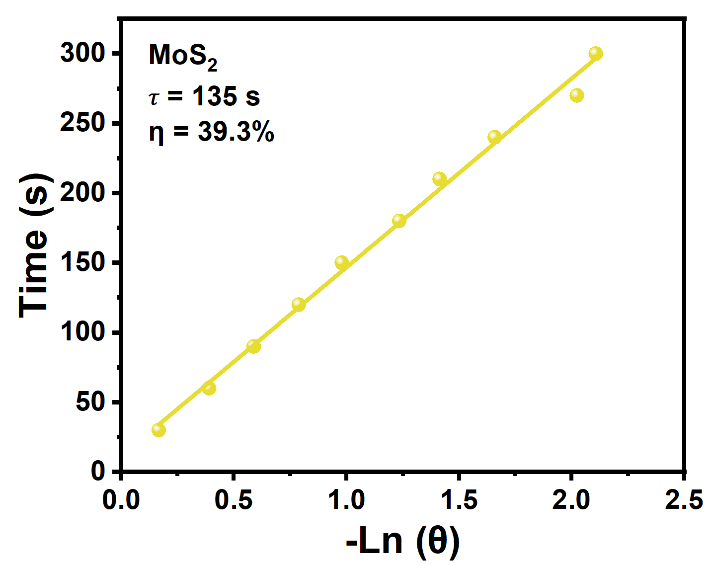


**Figure S7.** The photothermal conversion efficiency obtained from the relationship of linear time data vs −Ln θ.

**
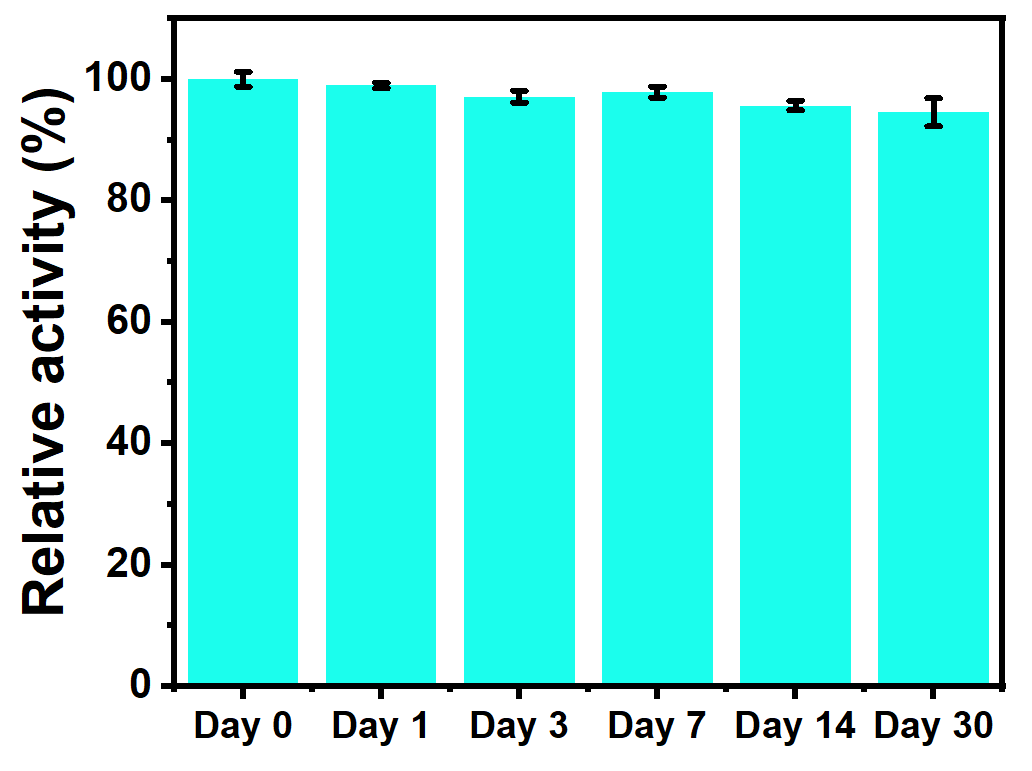
**

**Figure S8.** Effect of storage time on the POD-mimic catalytic performance during 30 days.

**
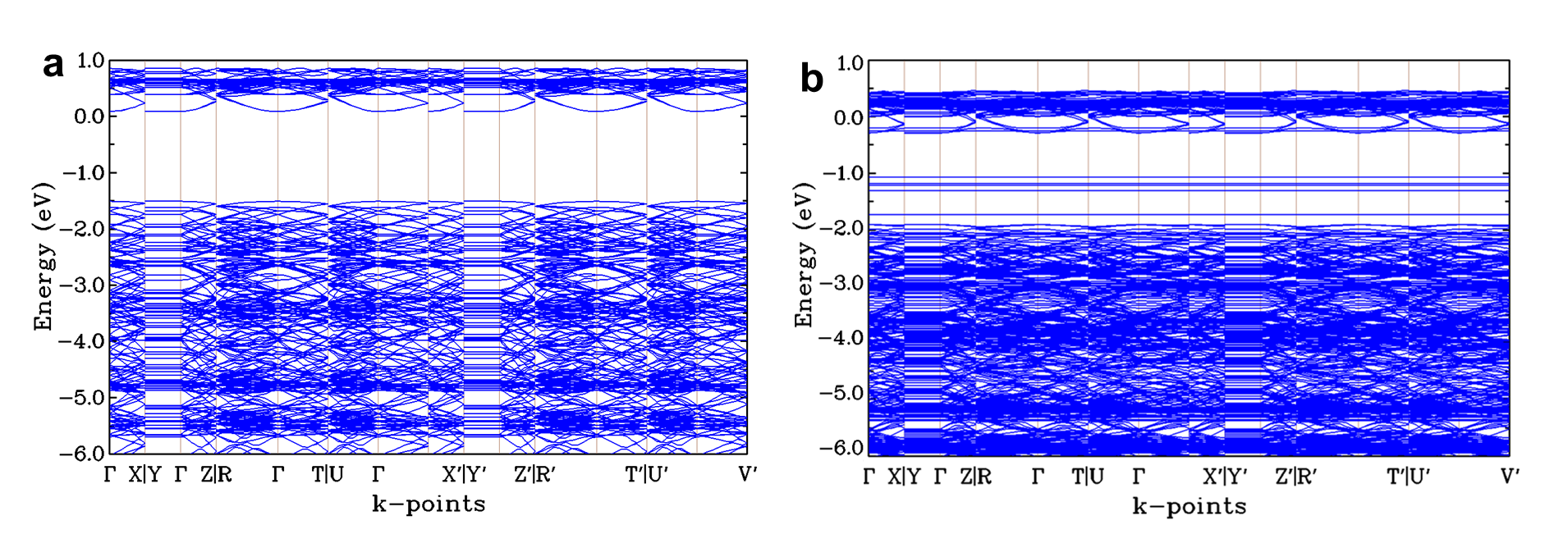
**

**Figure S9.** Band gap of the valence band maximum (VBM) and conduction band minimum (CBM) of (a) MoS_2_ and (b) Pt@MoS_2_.


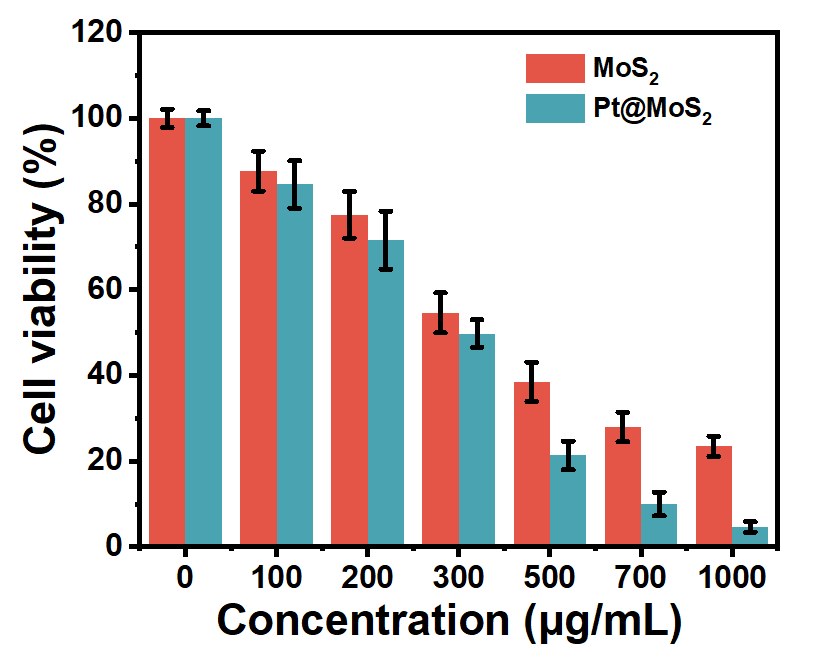


**Figure S10.** Cell viabilities after treatment with different concentrations of nanozymes.


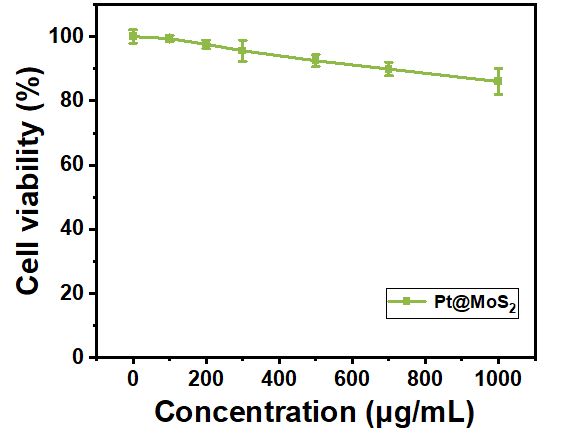


**Figure S11.** MIHA cell viabilities after treatment with different concentrations of Pt@MoS_2_ nanozymes.


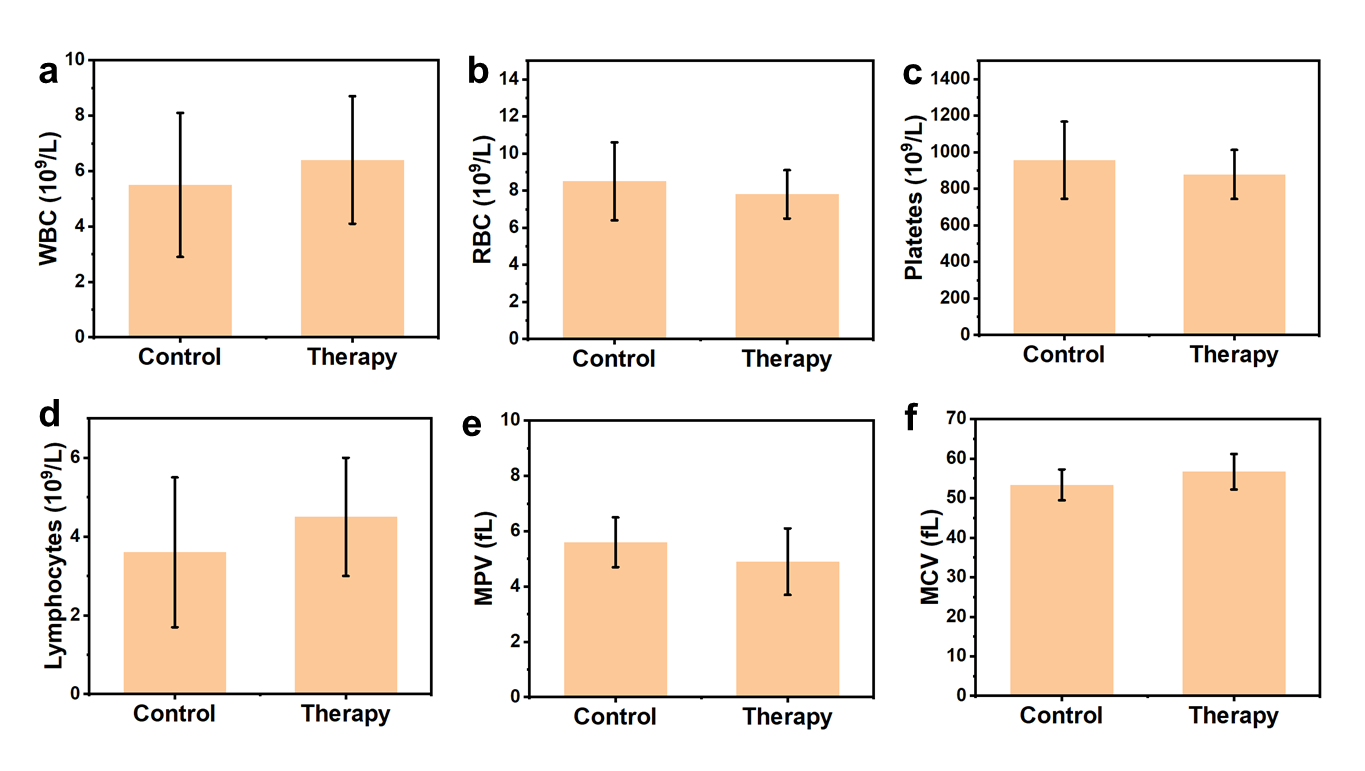


**Figure S12.** Routine blood examination analysis of tumor-bearing mice before and after therapy at 21th day. (a) white blood cells (WBC), (b) red blood cells (RBC), (c) platelets, (d) lymphocytes, (e) mean platelet volume (MPV), and (f) mean corpuscular volume (MCV).


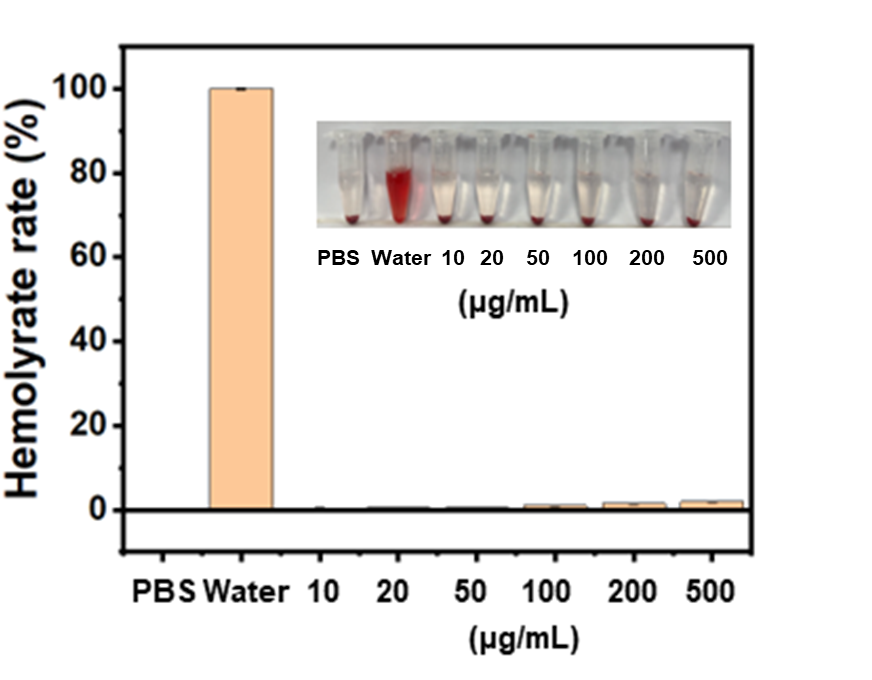


**Figure S13.** Blood compatibility of Pt@MoS₂


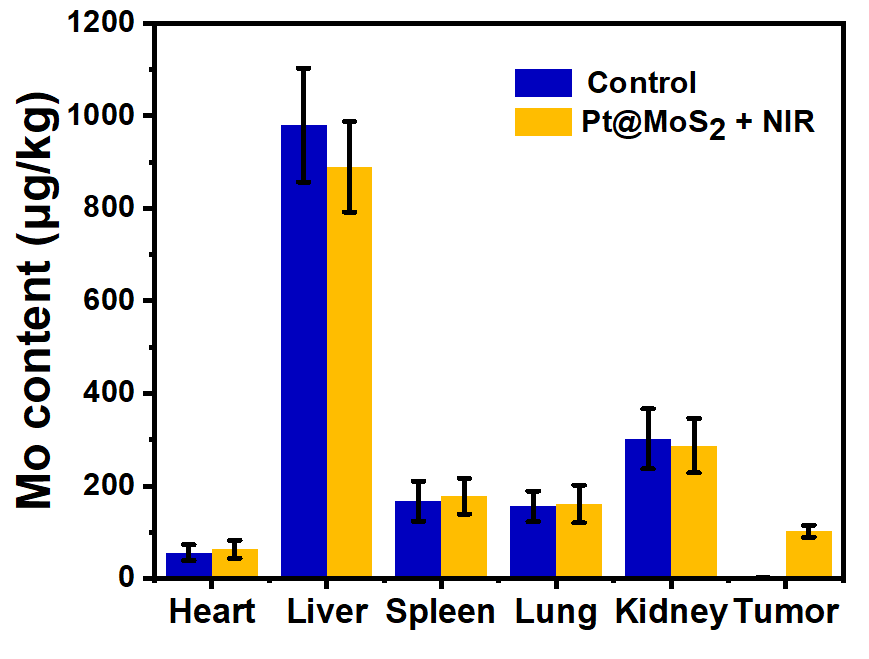


**Figure S14.** ICP-OES analysis of Mo element in different organs of control and Pt@MoS_2_+NIR groups after 21 days’ treatment.

**Table S1** Catalysts composition determined by ICP-OES.

| **Material** | **Mo (mol%)** | **Pt (mol%)** |
| --- | --- | --- |
| Pt@MoS_2_ | 94.2 | 5.8 |

Table S2. Comparison of K_m_ and V_max_ value of POD nanozymes

| Enzyme | K_m_, H_2_O_2_  (mM) | V_max_×10^-8^  （M S ^-1^） | Ref. |
| --- | --- | --- | --- |
| HRP | 3.70 | 8.71 | ^[1]^ |
| Fe_3_O_4_ | 154 | 9.78 | ^[1]^ |
| MoS_2_ | 2.812 | 8.01 | ^[2]^ |
| N-doped MoS_2_ | 0.4459 | 4.348 | ^[3]^ |
| La-RhRu | 0.72 | 23.4 | ^[4]^ |
| Fe_3_O_4_@MoS_2_-1%Ag | 1 | 18.2 | ^[5]^ |
| MoS_2_ | 0.54 | 4.51 | ^This work^ |
| Pt@MoS_2_ | 0.46 | 22.92 | ^This work^ |

Table S3. Comparison of K_m_ and V_max_ value of GSHOx nanozymes

| Enzyme | K_m_, GSH  (mM) | V_max_×10^-8^  （M S ^-1^） | Ref. |
| --- | --- | --- | --- |
| COFFePc | 9.78 | 81.7 | ^[6]^ |
| FeCo/Fe–Co | 1.775 | 107 | ^[7]^ |
| CDs@Pt | 1.04 | 746 | ^[8]^ |
| Cu_x_O@Bi_2_MoO_6_ | 0.606 | 14 | ^[9]^ |
| Bi_2_Fe_4_O_9_ | 1.85 | 92 | ^[10]^ |
| CuGQD/Pd@PSi | 0.4662 | 50.7 | ^[11]^ |
| MoS_2_ | 0.058 | 8.48 | ^This work^ |
| Pt@MoS_2_ | 0.053 | 17.98 | ^This work^ |

**References**

[1] L. Gao, J. Zhuang, L. Nie, J. Zhang, Y. Zhang, N. Gu, T. Wang, J. Feng, D. Yang, S. Perrett, Intrinsic peroxidase-like activity of ferromagnetic nanoparticles, Nature nanotechnology 2(9) (2007) 577-583.

[2] W. Yin, J. Yu, F. Lv, L. Yan, L.R. Zheng, Z. Gu, Y. Zhao, Functionalized nano-MoS2 with peroxidase catalytic and near-infrared photothermal activities for safe and synergetic wound antibacterial applications, ACS nano 10(12) (2016) 11000-11011.

[3] L. Feng, L. Zhang, S. Zhang, X. Chen, P. Li, Y. Gao, S. Xie, A. Zhang, H. Wang, Plasma-assisted controllable doping of nitrogen into MoS2 nanosheets as efficient nanozymes with enhanced peroxidase-like catalysis activity, ACS applied materials & interfaces 12(15) (2020) 17547-17556.

[4] J. Wu, Q. Liu, D. Jiao, B. Tian, Q. Wu, X. Chang, H. Chu, S. Jiang, Q. Yang, T. Liu, Tensile Strain‐Mediated Bimetallene Nanozyme for Enhanced Photothermal Tumor Catalytic Therapy, Angewandte Chemie International Edition (2024) e202403203.

[5] F. Wei, X. Cui, Z. Wang, C. Dong, J. Li, X. Han, Recoverable peroxidase-like Fe3O4@ MoS2-Ag nanozyme with enhanced antibacterial ability, Chemical Engineering Journal 408 (2021) 127240.

[6] M. Rong, J. Liu, L. Lu, Self‐Assembly of 2D Polyphthalocyanine in Lysosome Enables Multienzyme Activity Enhancement to Induce Tumor Ferroptosis, Advanced Healthcare Materials (2024) 2400325.

[7] Y. Liu, R. Niu, R. Deng, S. Song, Y. Wang, H. Zhang, Multi-enzyme co-expressed dual-atom nanozymes induce cascade immunogenic ferroptosis via activating interferon-γ and targeting arachidonic acid metabolism, Journal of the American Chemical Society 145(16) (2023) 8965-8978.

[8] L. Zhang, Q. Dong, Y. Hao, Z. Wang, W. Dong, Y. Liu, Y. Dong, H. Wu, S. Shuang, C. Dong, Drug‐Primed Self‐Assembly of Platinum‐Single‐Atom Nanozyme to Regulate Cellular Redox Homeostasis Against Cancer, Advanced Science 10(30) (2023) 2302703.

[9] G. Zhang, S. He, J. Wei, P. Ran, H. Zheng, L. He, X. Li, Interface‐Engineered CuxO@ Bi2MoO6 Heterojunctions to Inhibit Piezoelectric Screening Effect and Promote Double‐Nanozyme Catalysis for Antibacterial Treatment, Small 21(2) (2025) 2407281.

[10] Y. Zou, B. Jin, H. Li, X. Wu, Y. Liu, H. Zhao, D. Zhong, L. Wang, W. Chen, M. Wen, Cold nanozyme for precise enzymatic antitumor immunity, ACS nano 16(12) (2022) 21491-21504.

[11] J. Zhao, W. Duan, X. Liu, F. Xi, J. Wu, Microneedle patch integrated with porous silicon confined dual nanozymes for synergistic and hyperthermia‐enhanced nanocatalytic ferroptosis treatment of melanoma, Advanced Functional Materials 33(47) (2023) 2308183.
